# Production of Atosiban’s Key Intermediate Pentapeptide: Synthetic Approaches to the Development of a Peptide Synthesis with Less Racemization and Simplifier Purification Process

**DOI:** 10.3390/molecules27061920

**Published:** 2022-03-16

**Authors:** Chunying Ma, Yixuan Zheng, Jinwei Liu, Xiaobao Li, Wenhua Feng

**Affiliations:** Department of New Drug Research and Development, Institute of Materia Medica, Peking Union Medical College & Chinese Academy of Medical Sciences, Beijing 100050, China; machunying@imm.ac.cn (C.M.); chersnow@imm.ac.cn (Y.Z.); liujinwei20162016@163.com (J.L.); xiaobaoli@imm.ac.cn (X.L.)

**Keywords:** pentapeptide intermediate, atosiban, BSA/NHS, readily performed

## Abstract

The key intermediate NH_2_-Ile-Thr(Bzl)-Asn-Cys(Bzl)-Pro-COOH of Atosiban was prepared from *N*-Boc-*S*-Bzl-cysteine by the stepwise lengthening of the chain according to the repetitive *N*,*O*-bis(trimethylsilyl)acetamide/*N*-hydroxysuccinimide ester (BSA/NHS) strategy. This synthetic route required no chromatography purification and can be readily performed, yielding a highly pure pentapeptide compound.

## 1. Introduction

**Figure 1:**
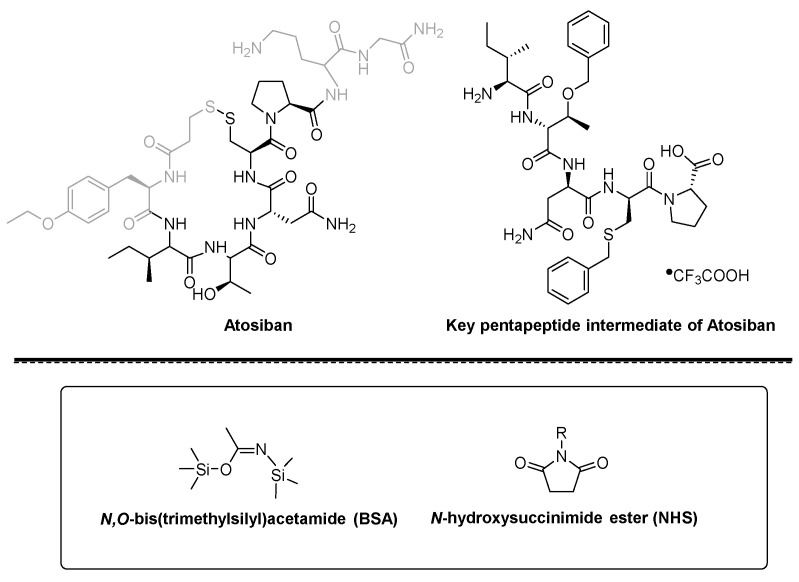
Structure of Atosiban, pentapeptide intermediate, BSA and NHS ester.

Atosiban (Figure 1) is a competitive antagonist of oxytocin receptors (OTR) that has been approved in Europe for the short-term treatment of preterm labor. It can inhibit oxytocin from binding to its receptors, which are expressed by the myoepithelial cells of the mammary gland, and in both the myometrium and endometrium of the uterus [1,2,3,4]. The solid-phase method has been widely adopted to synthesize Atosiban [5,6,7,8]; however, it is inappropriate to start large-scale productions due to its high cost. A solution synthesis route, which was accomplished by the addition of three polypeptide fragments end to end, was suggested for the preparation of Atosiban, and the pentapeptide NH_2_-Ile-Thr(Bzl)-Asn-Cys(Bzl)-Pro-COOH (Figure 1) was a key intermediate in the liquid synthesis [9].

However, most repetitive solution-phase methodologies used superstoichiometric amounts of a coupling reagent and activated amino acids to ensure high coupling efficiency, and later, the excess of the reagent had to be neutralized by additional reactions, followed by a purification procedure using several acidic and basic aqueous extractions [10,11,12,13]. These post-synthetic treatments caused the synthetic procedure to become complicated and time-consuming, and the severe conditions they used might have destroyed the peptide products and introduced undesirable impurities. In our previous study, a method using BSA/NHS (Figure 1) as coupling agents for the dipeptide synthesis was proven to be simple and efficient [14]. In order to further understand the BSA/NHS strategy in the synthesis of peptide, the key intermediate, NH_2_-Ile-Thr(Bzl)-Asn-Cys(Bzl)-Pro-COOH, was synthesized using the BSA/NHS strategy. In comparison, the synthesis of the key pentapeptide utilized five different coupling methods [15].

## 2. Results and Discussion

**Figure 2:**
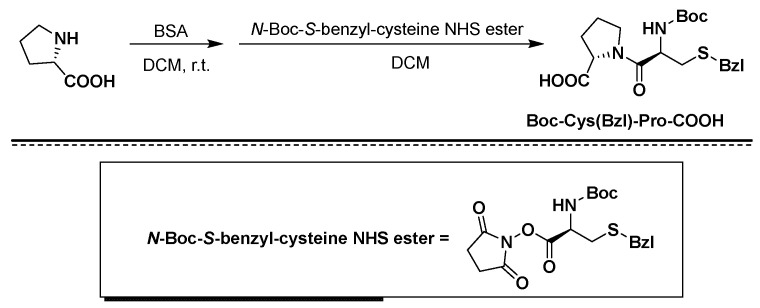
Synthesis of Boc-Cys(Bzl)-Pro-COOH using BSA/NHS as coupling agents.

Our previous experiments confirmed that unprotected amino acid could react with Boc-protected NHS ester with the assistance of BSA at room temperature in a high coupling efficiency for dipeptide synthesis [14]. In our current study, Boc-Cys(Bzl)-Pro-COOH was obtained using this approach (Figure 2) in a 92.4% yield.

**Figure 3:**
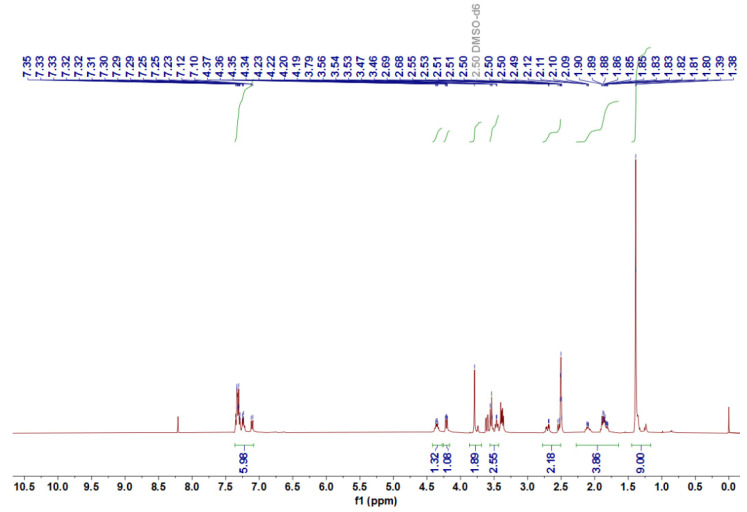
NMR analysis of Boc-Cys(Bzl)-Pro-COOH synthesized on BSA/NHS strategy.

In addition, in the synthesis of Atosiban’s key intermediate pentapeptide, all the excessive reagents and increased byproducts could be removed just using water or saturated sodium chloride solution rather than certain amounts of acidic and basic aqueous extractions [16]. All the excessive reactants, byproducts and the racemization products were undetected, according to the results of the NMR analysis (Figure 3).

**Figure 4:**
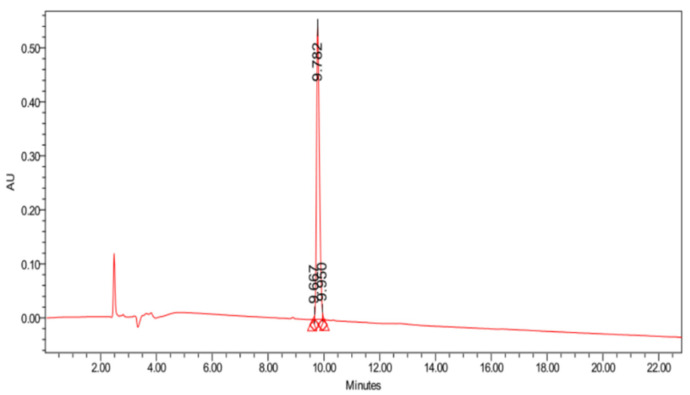
HPLC analysis of NH_2_-Cys(Bzl)-Pro-COOH.

The N-Boc protecting group was subsequently cleaved using trifluoroacetic acid/dichloromethane (TFA/CH_2_Cl_2_) (1:1), and the pure deprotected dipeptide NH_2_-Cys(Bzl)-Pro-COOH was obtained after the additional recrystallization from diethyl ether (94.0% yield). More impurities would be observed when just trifluoroacetic acid was utilized as a deprotection reagent, and therefore the yield would be low as well as the purification process would be more complicated. When peptide was produced in the form of hydrochloride salt, it would be more hygroscopic. According to the results of the HPLC analysis (Figure 4), no epimerization happened during the deprotection process either. Above all, we could synthesis dipeptide products in good yield and high purity in significantly shorter reaction times and with a simple purification process.

**Scheme 1:**
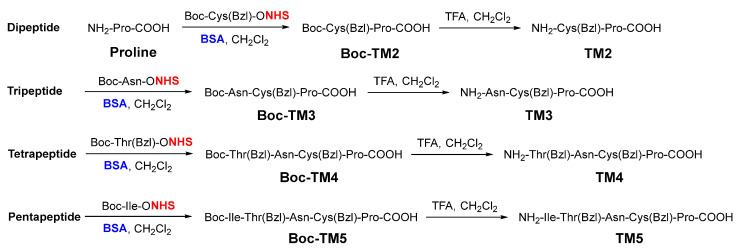
Solution-phase synthesis of NH_2_-Ile-Thr(Bzl)-Asn-Cys(Bzl)-Pro-COOH using repetitive BSA/NHS strategy.

Then, the peptide sequence could be extended by repeating the same coupling and deprotection cycle after the removal of the Boc-protecting group. The synthetic routine for the NH_2_-Ile-Thr(Bzl)-Asn-Cys(Bzl)-Pro-COOH was demonstrated in Scheme 1.

Before the synthesis of the pentapeptide intermediate, the N-Boc-protected amino acid fragments, including N-Boc-S-benzyl-cysteine (Cys), N-Boc-asparagine (Asn), N-Boc-O-benzyl threonine (Thr) and N-Boc-isoleucine (Ile), were derivatized with NHS ester at the C-terminus in advance [17].

**Figure 5:**
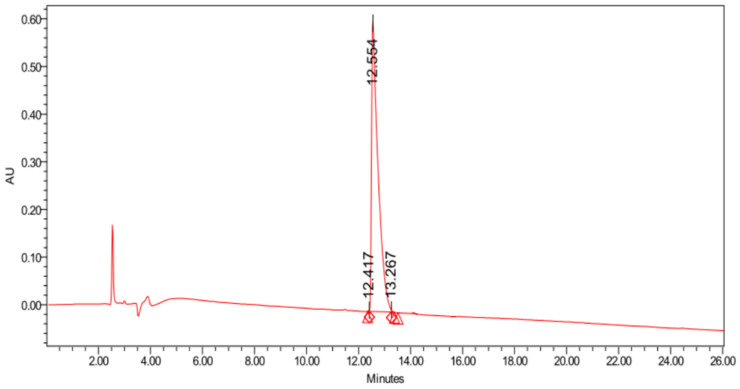
HPLC analysis of NH_2_-Ile-Thr(Bzl)-Asn-Cys(Bzl)-Pro-COOH synthesized on BSA/NHS strategy.

Based on the above study, the deprotected dipeptide **TM2** was obtained in good yield as colorless bulk crystals. Then, the reaction between **TM2** and Boc-Asn-ONHS afforded the Boc-protected tripeptide Boc-**TM3** in 92.1% yield, which was recrystallized from ethyl acetate. The Boc group was then cleaved and **TM3** precipitated as a white solid after the addition of anhydrous diethyl ether (93.7% yield). The resulting peptide **TM3** reacted with Boc-Thr(Bzl)-ONHS to produce Boc-**TM4** in 91.2% yield. After the same deprotection steps as above, the deprotected tetrapeptide **TM4** was reacted with Boc-Ile-ONHS to give Boc-**TM5** after recrystallization from ethyl acetate/diethyl ether in the yield of 88.6%. After the same deprotection steps, the final product, **TM5**, was obtained in 90.9% yield. Both the deprotected tetrapeptide **TM4** and deprotected pentapeptide **TM5** were recrystallized from anhydrous diethyl ether. The HPLC analysis of the deblocked pentapeptide, **TM5**, demonstrated that even after four coupling cycles, no further purification was required (Figure 5).

## 3. Materials and Methods

All raw materials, reagents and solvents were purchased from commercial suppliers and used without further purification. NMR spectra were acquired in chloroform (CDCl_3_), methanol-*d*_4_ and dimethylsulfoxide-*d*_6_ (DMSO-*d*_6_) using a Varian Inova 400 (400 MHz) instrument. The tetramethylsilane, as a reference, was used.

Chromatographic conditions: Instrument: Waters 2695; chromatographic column: Diamonsil C18 (5 uM, 250 * 4.6 mm); flow rate: 1 mL/min; column temperature: 30 °C; mobile phase: phase A: CH_3_CN, 0.1% TFA; phase B: water, 0.1% TFA; gradient conditions: 0.01 min → 25.0 min: 5% phase A → 70% phase A; 25.0 min → 30 min: 70% phase A → 90% phase A.

General Procedure for the formation of amide bond using BSA/NHS strategy. Under argon protection, BSA (2.2 equiv.) was added to amino precursor (1.1 equiv.) in anhydrous CH_2_Cl_2_. After the mixture was stirred for 1–24 h at 25 °C, a solution of *N*-Boc protected NHS ester (1 equiv.) in dichloromethane was added. The reaction mixture was stirred at 25 °C under argon until all active ester was consumed, as judged by the TLC analysis. The reaction mixture was washed with brine, dried over Na_2_SO_4_ and concentrated in vacuo to provide a white solid. The isolated product was recrystallized from diethyl ether/n-hexane to yield the targeted compound.

General Procedure for the Boc-deprotected reaction. The material was dissolved in CH_2_Cl_2_ and a solution of TFA/CH_2_Cl_2_ (1:2) (10 equiv.) was added. After the mixture was stirred for 4 h at 25 °C, the reaction mixture was concentrated in vacuo to yield a yellow oil. Afterwards, the pure product was recrystallization from diethyl ether as a white solid.

## 4. Conclusions

In summary, we have successfully developed a rapid, large-scale solution-phase synthesis of the key pentapeptide intermediate of Atosiban in a repetitive BSA/NHS strategy. Less racemization happened, shorter numbers of unit operation were necessary and the purification process was more simplified than other repetitive solution-phase methodologies. Above all, the repetitive BSA/NHS strategy has the potential to be applied in the further commercial-scale manufacturing of more peptide drugs.

## Data Availability

Data are contained within the article or the Supplementary Material.

## Supplementary Materials

The following supporting information can be downloaded at https://www.mdpi.com/article/10.3390/molecules27061920/s1. Figure S1: The ^1^H NMR of Boc-Cys(Bzl)-ONHS; Figure S2: The ^13^C NMR of Boc-Cys(Bzl)-ONHS; Figure S3: The ^1^H NMR of Boc-Asn-ONHS; Figure S4: The ^13^C NMR of Boc-Asn-ONHS; Figure S5: The ^1^H NMR of Boc-Thr(Bzl)-ONHS; Figure S6: The ^13^C NMR of Boc-Thr(Bzl)-ONHS; Figure S7: The ^1^H NMR of Boc-Ile-ONHS; Figure S8: The ^13^C NMR of Boc-Ile-ONHS; Figure S9: The ^1^H NMR of Boc-**TM2**; Figure S10: The ^13^C NMR of Boc-**TM2**; Figure S11: The ^1^H NMR of **TM2**; Figure S12: The ^13^C NMR of **TM2**; Figure S13: The ^1^H NMR of Boc-**TM3**; Figure S14: The ^13^C NMR of Boc-**TM3**; Figure S15: The ^1^H NMR of **TM3**; Figure S16: The ^13^C NMR of **TM3**; Figure S17 The MS spectrum of **TM3**; Figure S18: The ^1^H NMR of Boc-**TM4**; Figure S19: The ^13^C NMR of Boc-**TM4**; Figure S20: The ^1^H NMR of **TM4**; Figure S21: The ^13^C NMR of **TM4**; Figure S22: The MS spectrum of **TM4**; Figure S23: The ^1^H NMR of Boc-**TM5**; Figure S24: The ^13^C NMR of Boc-**TM5**; Figure S25: The ^1^H NMR of **TM5**. Figure S26: The ^13^C NMR of **TM5**; Figure S27: The MS spectrum of **TM5**.

## Sample Availability

Samples of the compounds are not available from the authors.
