# Supplementary material for: Production of Atosiban’s Key Intermediate Pentapeptide: Synthetic Approaches to the Development of a Peptide Synthesis with Less Racemization and Simplifier Purification Process
Source: Molecules. 2022 Mar 16;27(6):1920. doi: 10.3390/molecules27061920 (PMC8951825; doi:10.3390/molecules27061920)
Supplement: Supplementary file 1 [file molecules-27-01920-s001.zip › molecules-1534673-supplementary.pdf]

Production of Atosiban's key intermediate pentapeptide: Synthetic approaches to the development of a rapid solution-phase-peptide synthesis with less racemization and simplifier purification process

Chunying Ma, Yixuan Zheng, Jinwei Liu, XiaoBao Li, Wenhua Feng*

Department of New Drug Research and Development, Institute of Materia Medica, Peking Union Medical College & Chinese Academy of Medical Sciences, Beijing, China.

Table of Contents

General procedure for preparation of *N*-hydroxysuccinimide ester of amino acid …….. ……… 2

Preparation of *N*-Boc-*S*-benzyl-*L*-cysteine-*N*-succinimidyl ester (Boc-Cys(Bzl)-ONHS) …………..2

Preparation of *N*-Boc-*L*-Asparagine-*N*-succinimidyl ester (Boc-Asn-ONHS) ………….. ………….. 2

Preparation of *N*-Boc-*O*-benzyl-*L*-threonine-*N*-succinimidyl ester (Boc-Thr(Bzl)-ONHS) …………2

Preparation of Boc-*L*-Isoleucine-*N*-succinimidyl Ester (Boc-Ile-ONHS) ………….. ………….. …… 3

Preparation of Boc-Cys(Bzl)-Pro-COOH (Boc-TM2) ………….. ………….. ………. ………….. ………3

Preparation of *L*-Cys(Bzl)-Pro-OH*CF_3COOH (TM2) ………….. ………….. ………. ………….. ………4

Preparation of Boc-Asn-L-Cys(Bzl)-Pro-OH (Boc-TM3) ………….. ………….. ………. ………….. … 4

Preparation of Asn-*L*-Cys(Bzl)-Pro-OH*CF_3COOH (TM3)………. ………….. ………. …………. …. 4

Preparation of Boc-Thr(Bzl)-Asn-*L*-Cys(Bzl)-Pro-OH (Boc-TM4) .. ………….. ………. ………….. …5

Preparation of Thr(Bzl)-Asn-L-Cys(Bzl)-Pro-OH*CF_3COOH (TM4) . ………….. ………. ………….. …5

Preparation of Boc-Ile-Thr(Bzl)-Asn-L-Cys(Bzl)-Pro-OH (Boc-TM5) ………. ………. ………….. 5

Preparation of H-Ile-Thr(Bzl)-Asn-L-Cys(Bzl)-Pro-OH*CF_3COOH (TM5) ………. ………. ………….. 6

^{1}H NMR, ^{13}C NMR spectra and the MS spectra ………. ………….. ………. ………….. ……………7-20

General procedure for preparation of *N*-hydroxysuccinimide ester of amino acid. *N, N*-Dicyclohexylcarbodiimide (1.1 equiv.) was added to a solution of amino acid (1.0 equiv.) and *N*-hydroxysuccinimide (1.1 equiv.) in dioxane. The reaction mixture was stirred and kept at 5°C for a period of 15 h. The formed dicyclohexyl urea was filtered and washed with dioxane, and the filtrate was concentrated in vacuo to yield a yellow oil. The oil was recrystallized to yield the analytically pure product.

Preparation of *N*-Boc-*S*-benzyl-*L*-cysteine-*N*-succinimidyl ester (Boc-Cys(Bzl)-ONHS).

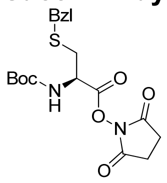

N-hydroxysuccinimide (2.19 g, 18.72 mmol) was added to a solution of *N*-*tert*-butyloxycarboryl-*S*-benzyl-*L*-cysteine (6 g, 19.26 mmol) in 10 mL of anhydrous 1,4-dioxane at 25°C. The reaction mixture was cooled to 0°C and then a solution of DCC (4.35 g, 21.18 mmol) in 3 mL of dichloromethane (CH_2Cl_2) was added slowly and stirred at 5°C for 15 h. After filtration, the solution was removed under reduced pressure. The isolated product was recrystallized from isopropanol to yield **Boc-Cys(Bzl)-ONHS** as a white solid (6.06 g, 77.0%). ^{1}H NMR ($CDCl_3$) *δ*/ppm=1.49 (s, 9H), 2.81 (s, 4H), 2.9 (m, 2H), 3.80 (s, 2H), 4.80 (m, 1H), 5.24 (d, 1H, *J*=6.7Hz) and 7.32 (m, 5H). ^{13}C NMR (100 MHz, $CDCl_3$) *δ*/ppm=171.9, 168.6, 167.3, 154.9, 137.5, 129.2, 128.8, 127.5, 80.9, 77.4, 63.8, 51.7, 36.8, 33.8, 28.4, 25.7 and 25.5.

Preparation of *N*-Boc-*L*-Asparagine-*N*-succinimidyl ester (Boc-Asn-ONHS)

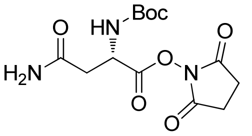

N-hydroxysuccinimide(1.5 g, 12.93 mmol) was added to a solution of *N*-*tert*-butyloxycarboryl-*L*-asparagine (3.0 g, 12.93 mmol) in 10 mL of anhydrous 1,4-dioxane at 25°C. The reaction mixture was cooled to 0°C, then a solution of DCC (2.67 g, 12.95 mmol) in 3 mL of CH_2Cl_2 was added slowly and stirred at 5°C for 15h. After filtration, the solution was removed under reduced pressure. Another 8 mL of CH_2Cl_2 was added and the mixture was filtrated again. After the CH_2Cl_2 was removed under reduced pressure, the isolated product was recrystallized from methanol to yield **Boc-Asn-ONHS** as a white solid (3.12 g, 73.3%). ^{1}H NMR (400 MHz, DMSO-d_6) *δ*/ppm=1.38(s, 9H), 2.61(m, 2H), 2.79(s, 4H), 4.74(m, 1H), 7.01 (s, 1H), 7.43(s, 1H) and 7.51 (d, 2H, *J*=10 Hz). ^{13}C NMR (100 MHz, DMSO-d_6) *δ*/ppm=170.4, 170.3, 167.0, 155.4, 79.2, 49.0, 36.8, 28.6, 25.9 and 25.7.

Preparation of *N*-Boc-*O*-benzyl-*L*-threonine-*N*-succinimidyl ester (Boc-Thr(Bzl)-ONHS)

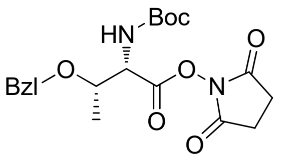

N-hydroxsuccinimide (1.62 g, 14.19 mmol) was added to a solution of *N*-*tert*-butyloxycarboryl-*O*-benzyl-*L*-threonine (4.38 g, 14.19 mmol) in 10 mL of anhydrous 1,4-dioxane at 25°C. The reaction mixture was cooled to 0°C, then a solution of DCC (2.94 g, 14.19 mmol) in 6 mL of CH_2Cl_2 was added slowly and stirred at 5°C for 15h. After filtration, the solution was removed under reduced pressure. Another 18 mL of CH_2Cl_2 was added and the mixture was filtrated again. After the CH_2Cl_2 was removed under reduced pressure, the isolated product was recrystallized from ethyl acetate/petroleum ether (4/1) to yield **Boc-Thr(Bzl)-ONHS** as a white solid (4.89 g, 84.8%). ^{1}H NMR (400 MHz, $CDCl_3$) *δ*/ppm=1.31 (d, 3H, *J*=6.4 Hz), 1.45 (s, 9H), 2.85 (s, 4H), 4.30 (q, 1H, *J*=6.8 Hz), 4.56 (dd, 2H, *J*=5.8 Hz, 12.8Hz), 4.71 (d, 1H, *J*=8.7Hz), 5.35 (d, 1H, *J*=8.6Hz) and 7.30 (m, 5H). ^{13}C NMR (100 MHz, $CDCl_3$) *δ*/ppm=170.9, 168.9, 166.8, 155.4, 137.6, 128.0, 127.9, 127.7, 127.5, 80.0, 74.6, 71.4, 60.0, 27.9, 27.6, 25.3, 25.0 and 20.6.

Preparation of Boc-*L*-Isoleucine-*N*-succinimidyl Ester (Boc-Ile-ONHS)

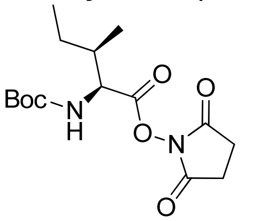

N-hydroxysuccinimide (1.44 g, 12.48 mmol) was added to a solution of *N*-*tert*-butyloxycarboryl-*L*-isoleucine (2.88 g, 12.48 mmol) in 10 mL of anhydrous 1,4-dioxane at 25°C. The reaction mixture was cooled to 0°C, and then a solution of DCC (2.64 g, 12.75 mmol) in 3 mL of CH_2Cl_2 was added slowly and stirred at 5°C for 15 h. After filtration, the solution was removed under reduced pressure. Another 6 mL of CH_2Cl_2 was added and the mixture was filtrated again. After the CH_2Cl_2 was removed under reduced pressure, the isolated product was recrystallized from ethyl acetate to yield **Boc-Ile-ONHS** as a white solid (2.61 g, 63.7%). ^{1}H NMR (400 MHz, $CDCl_3$) *δ*/ppm=0.97 (t, 3H, *J*=7.4Hz), 1.04 (d, 3H, *J*=6.7Hz), 1.26-1.33 (m, 1H), 1.57 (s, 9H), 1.59 (m, 1H), 1.92 (m, 1H), 2.90 (s, 4H), 4.63 (m, 1H) and 5.01 (d, 1H, *J*=8.6Hz). ^{13}C NMR (100 MHz, $CDCl_3$) *δ*/ppm=169.0, 167.6, 155.0, 79.8, 56.3, 37.5, 27.9, 27.6, 25.3, 24.4, 14.7 and 13.8.

Preparation of Boc-Cys(Bzl)-Pro-COOH (Boc-TM2)

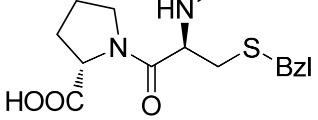

Under argon protection, BSA (3.04 mL, 12.42 mmol) was added dropwise to a solution of proline (0.6 g, 5.17mmol) in 10 mL of CH_2Cl_2 at 0°C. After the mixture was stirred for 2 h at 25°C, a solution of **Boc-Cys(Bzl)-ONHS** (2.11 g, 5.17 mmol) in 2 L of CH_2Cl_2 was added dropwise. The reaction mixture was stirred until all active ester was consumed, as judged by the TLC analysis (reaction time: 12h) at

25°C under argon. The reaction mixture was washed with brine, dried over Na_2SO_4 and concentrated in vacuo to provide a white solid. The isolated product was recrystallized from ethyl acetate/diethyl ether (1:1) to yield **Boc-TM2** as a white solid of 2.0 g (92.4%). ESI-MS (*m*/*z*): 409.3 $[M+H]^+$. ^{1}H NMR (400 MHz, DMSO-*d*6), *δ*/ppm=1.38 (s, 9H), 1.80-2.12 (m, 4H), 2.49-2.69 (m, 2H), 3.46-3.56 (m, 2H), 3.79 (s, 2H), 4.21 (dd, 1H, *J*=4Hz, 12Hz), 4.34-4.37 (m, 1H) and 7.10-7.35 (m, 5H). ^{13}C NMR (100 MHz, DMSO-*d*6) *δ*/ppm=173.5, 168.9, 155.3, 138.4, 129.0, 128.4, 126.9, 78.2, 63.9, 58.9, 51.8, 46.3, 32.4, 28.6, 28.2 and 24.5.

Preparation of *L*-Cys(Bzl)-Pro-OH*CF_3COOH (TM2)

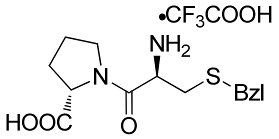

A solution of TFA (3.4 mL) in CH_2Cl_2 (3.4 mL) was added dropwise to a solution of **Boc-TM2** (2.0 g, 4.89 mmol) in CH_2Cl_2 (3.4 mL) at 0ºC. After the reaction mixture was stirred at room temperature for 4 h, the solvent was removed under reduced pressure to yield a yellow oil. Then, diethyl ether (3 mL) was added and white solid precipitated. After filtration, 2.40 g (94.0%) of the compound, **TM2**, was obtained. ESI-MS (m/z): 309.3 $[M+H-CF_3COOH]^+$. ^{1}H NMR (400 MHz, DMSO-*d*6) *δ*/ppm= 1.83-1.93 (m, 3H), 2.17-2.22 (m, 1H), 2.72 (dd, 1H, *J*=8Hz, 12Hz), 2.88 (dd, 1H, *J*=8Hz, 12Hz), 3.36-3.42 (m, 1H), 3.66-3.70 (m, 1H), 3.90 (s, 2H), 4.28-4.35 (m, 2H) and 7.24-7.40 (m, 5H). ^{13}C NMR (100 MHz, DMSO-*d*6) *δ*/ppm=173.0, 166.6, 138.2, 129.6, 128.9, 127.6, 59.4, 50.7, 47.2, 35.5, 31.4, 29.1 and 25.0.

Preparation of Boc-Asn-L-Cys(Bzl)-Pro-OH (Boc-TM3)

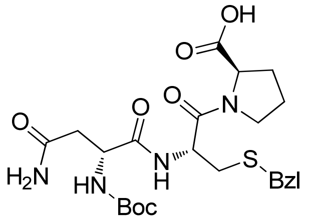

Under argon protection, BSA (2.67 mL, 10.96 mmol) was added dropwise to a solution of **TM2** (1.64 g, 5.03 mmol) in 10 mL of dichloromethane at 0°C. After the mixture was stirred for 2 h at 25°C, a solution of **Boc-Asn-ONHS** (1.93 g, 4.58 mmol) in 2 L of dichloromethane was added dropwise. The reaction mixture was stirred until all active ester was consumed, as judged by the TLC analysis (reaction time: 14 h) at 25°C under argon. The reaction mixture was washed with brine, dried over Na_2SO_4 and concentrated in vacuo to provide a white solid. The isolated product was recrystallized from ethyl acetate/diethyl ether to yield **Boc-TM3** as a white solid of 2.2 g (92.1%). ESI-MS (*m*/*z*): 523.5 $[M+H]^+$. ^{1}H NMR (DMSO-*d*6) *δ*/ppm=1.37 (s, 9H), 1.84-1.90 (m, 3H), 2.07-2.14 (m, 1H), 2.31-2.37 (m, 1H), 2.43-2.53 (m, 3H), 2.74-2.97 (m, 1H), 3.42-3.57 (m, 2H), 3.72-3.82 (m, 2H), 4.18-4.28 (m, 2H), 4.64-4.69 (m, 1H), 6.88-6.94 (m, 2H) and 7.20-7.35 (m, 5H). ^{13}C NMR (100 MHz, DMSO-*d*6) *δ*/ppm=173.0, 171.4, 171.4, 168.4, 155.2, 138.4, 129.0, 128.9, 128.4, 128.3, 126.8, 78.3, 58.7, 51.4, 50.3, 46.5, 37.2, 35.4, 32.3, 28.6, 28.2 and 24.4.

Preparation of Asn-*L*-Cys(Bzl)-Pro-OH*CF_3COOH (TM3)

A solution of TFA (3.6 mL) in CH_2Cl_2 (3.6 mL) was added dropwise to a solution of **Boc-TM3** (2.2 g, 4.22 mmol) in CH_2Cl_2 (4 mL) at 0°C. After the reaction mixture was stirred at room temperature for 4 h, the solvent was removed under reduced pressure to yield a yellow oil. Then, diethyl ether (4 mL) was added and white solid precipitated. After filtration, 2.1 g (93.7%) of the compound, **TM3**, was obtained. ESI-MS (*m*/*z*): 421.2 $[M-H-CF_3COOH]^-$. ^1H NMR (400 MHz, DMSO-d_6) *δ*/ppm=1.86-1.89(m, 3H), 2.11-2.16 (m, 1H), 2.50-2.59 (m, 2H), 3.40-3.57 (m, 2H), 3.81 (s, 2H), 4.07-4.22 (m, 2H), 4.62-4.64 (m, 1H) and 7.22-7.37 (m, 5H). ^{13}C-NMR (100 MHz, DMSO-*d*6) *δ*/ppm=173.4, 171.0, 168.6, 168.4, 138.8, 129.4, 128.9, 127.4, 59.3, 51.4, 49.4, 47.0, 36.0, 35.9, 32.3, 29.1 and 24.9.

Preparation of Boc-Thr(Bzl)-Asn-*L*-Cys(Bzl)-Pro-OH (Boc-TM4)

Under argon protection, BSA (2.39 mL, 0.98 mmol) was added dropwise to a solution of **TM3** (2.17 g, 4.02 mmol) in 10 mL of CH_2Cl_2 at 0°C. After the mixture was stirred for 3 h at 25°C, a solution of the **Boc-Thr(Bzl)-ONHS** (1.8 g, 4.45 mmol) in 2 mL of dichloromethane was added dropwise. The reaction mixture was stirred until all active ester was consumed, as judged by TLC analysis (reaction time: 20 h) at 25°C under argon. Then, the reaction mixture was washed with brine, dried over Na_2SO_4 and concentrated in vacuo to provide a white solid. The isolated product was recrystallized from ethyl acetate/diethyl ether to yield **Boc-TM4** as a white solid of 2.6 g (91.2%). ESI-MS (*m*/*z*): 714.5 $[M+H]^+$. ^1H NMR (400 MHz, CD_3OD) *δ*/ppm=1.19 (d, *J*=8Hz, 3H), 1.44 (s, 9H),1.94-1.96 (m, 3H), 2.17-2.19 (m, 1H), 2.50-2.55 (m, 1H), 2.67-2.70 (m, 2H), 2.78-2.84 (m, 1H), 3.39-3.42 (m, 1H), 3.57-3.60 (m, 1H), 3.73-3.74 (m, 2H), 4.02-4.03 (m, 1H), 4.19-4.21 (m, 1H), 4.34-4.37 (m, 1H), 4.48-4.59 (m, 2H) , 4.67-4.78 (m, 2H) and 7.20-7.36 (m, 10H). ^{13}C NMR (100 MHz, CD_3OD) *δ*/ppm=175.2, 174.8, 172.6, 170.7, 158.0, 139.7, 130.2, 130.1, 129.6, 129.5, 129.4, 129.2, 129.0, 128.7, 128.2, 128.0, 81.1, 76.2, 72.5, 60.6, 60.1, 52.1, 51.9, 51.4, 37.7, 37.1, 33.3, 30.2, 28.7, 25.6 and 16.6.

Preparation of Thr(Bzl)-Asn-L-Cys(Bzl)-Pro-OH*CF_3COOH (TM4)

A solution of TFA (3 mL) in CH_2Cl_2 (3 mL) was added dropwise to a solution of **Boc-TM4** (2.6 g, 3.64 mol) in CH_2Cl_2 (5 mL) at 0°C. After the reaction mixture was stirred at room temperature for 3 h, the solvent was removed under reduced pressure to yield a yellow oil. Then, diethyl ether (4 mL) was added and white solid precipitated. After filtration, 2.73 g (90.9%) of the compound, **TM4**, was obtained. ESI-MS(*m*/*z*): 614.3 $[M+H-CF_3COOH]^+$. ^1H NMR (400 MHz, DMSO-d_6) *δ*/ppm=1.23-1.24 (m, 3H), 1.82-1.88 (m, 3H), 2.07-2.11 (m, 1H), 2.44-2.74 (m, 4H), 3.39-3.54 (m, 2H), 3.74-3.86 (m, 4H), 4.16-4.19 (m, 1H), 4.46-4.56 (m, 2H), 4.61-4.70 (m, 2H) and 7.21-7.42 (m, 10H). ^{13}C-NMR (100 MHz, DMSO-d_6) *δ*/ppm= 173.5, 171.2, 170.9, 168.7, 166.9, 138.9, 138.5, 129.4, 129.0, 128.9, 128.8, 128.6, 128.4, 128.0, 127.4, 127.3, 74.4, 71.1, 59.2, 56.9, 51.4, 50.3, 46.9, 37.6, 35.9, 32.4, 29.1, 24.9 and 16.6.

Preparation of Boc-Ile-Thr(Bzl)-Asn-L-Cys(Bzl)-Pro-OH (Boc-TM5)

Under argon protection, BSA (2.02 mL, 8.37 mmol) was added dropwise to a solution of **TM4** (2.7 g,

3.78 mmol) in 10 mL of dichloromethane at 0°C. After the mixture was stirred for 3 h at 25°C, a solution of **Boc-Ile-ONHS** (1.08 g, 4.86 mmol) in 5 mL of CH_2Cl_2 was added dropwise. The reaction mixture was stirred until all active ester was consumed, as judged by the TLC analysis (reaction time 14h) at 25°C under argon. Then, the reaction mixture was washed with brine, dried over Na_2SO_4 and concentrated in vacuo to provide a white solid. The isolated product was recrystallized from ethyl acetate/diethyl ether to yield **Boc-TM5** as a white solid, 2.7 g (88.6%). ESI-MS (*m/z*): 827.4 $[M+H]^+$. ^1H NMR (400 MHz, CD_3OD) *δ*/ppm=0.86-0.92 (m, 6H), 1.19 (d, *J*=8Hz, 3H), 1.40 (s, 9H), 1.45-1.51 (m, 1H), 1.79-1.84 (m, 1H), 1.93-1.97 (m, 3H), 2.16-2.20 (m, 1H), 2.56-2.70 (m, 1H), 2.71-2.84 (m, 3H), 3.44-3.69 (m, 1H), 3.69-3.83 (m, 5H), 3.96 (d, *J*=8Hz, 1H), 4.03-4.06 (m, 1H), 4.34-4.37 (m, 1H), 4.50-4.60 (m, 3H), 4.69-4.83 (m, 2H) and 7.18-7.37 (m, 10H). ^{13}C NMR (100 MHz, CD_3OD) *δ*/ppm=175.2, 174.8, 174.5, 172.6, 170.7, 158.0, 139.7, 139.6, 130.2, 130.1, 129.6, 129.5, 129.4, 129.0, 128.7, 128.2, 128.0, 80.8, 75.8, 71.4, 65.1, 61.0, 60.5, 58.6, 52.1, 51.5, 37.9, 37.8, 37.1, 33.3, 30.2, 28.7, 25.9, 25.6, 16.7, 16.2 and 11.6.

Preparation of H-Ile-Thr(Bzl)-Asn-L-Cys(Bzl)-Pro-OH*CF_3COOH (TM5)

A solution of TFA (8 mL) in CH_2Cl_2 (8 mL) was added dropwise to a solution of **Boc-TM5** (2.72 g, 3.27 mmol) in CH_2Cl_2 (10 mL) at 0°C. After the reaction mixture was stirred at room temperature for 3 h, the solvent was removed under reduced pressure to yield a yellow oil. Then, diethyl ether (10 mL) was added and white solid precipitated. After filtration, 2.5 g (90.9%) of the compound, **TM5**, was obtained. ESI-MS(*m/z*): 725.5$[M-H-CF_3COOH]^-$. ^1H NMR (400 MHz, CD_3OD) *δ*/ppm=0.82-0.91 (m, 6H), 1.11-1.14 (m, 3H), 1.23-1.26 (m, 1H), 1.48-1.53 (m, 1H), 1.83-1.85 (m, 3H), 2.06-2.09 (m, 1H), 2.36-2.53 (m, 2H), 2.67-2.72 (m, 1H), 3.39-3.42 (m, 1H), 3.50-3.52 (m, 1H), 3.68-3.75 (m, 2H), 3.84-3.92 (m, 3H), 4.15-4.18 (m, 1H), 4.52 (s, 2H), 4.61-4.63 (m, 3H) and 7.27-7.36 (m, 10H). ^{13}C-NMR (100 MHz, CD_3OD) *δ*/ppm=173.5, 171.6, 171.1, 169.1, 168.6, 139.0, 138.9, 129.4, 129.3, 128.9, 128.8, 128.6, 128.2, 127.8, 127.4, 127.2, 75.4, 71.0, 59.2, 56.7, 56.5, 51.0, 50.1, 46.9, 37.7, 36.8, 35.9, 32.5, 29.1, 24.8, 24.2, 16.7, 15.1 and 11.5.

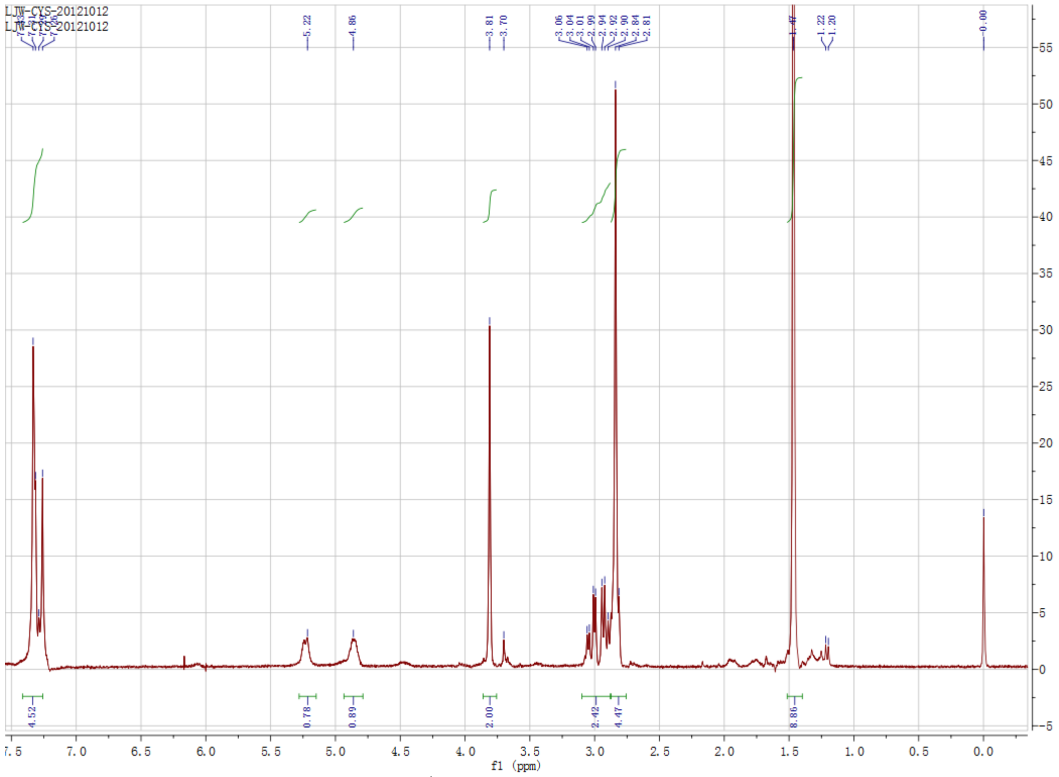

Figure S1. The ^{1}H NMR of **Boc-Cys(Bzl)-ONHS.**

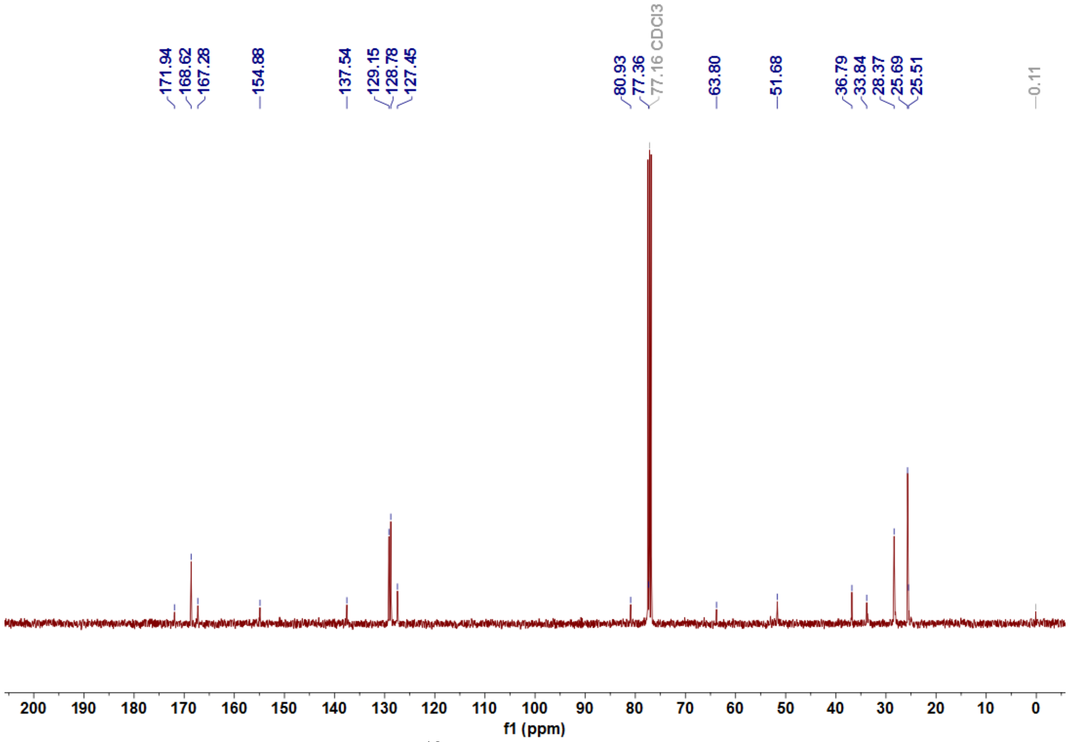

Figure S2. The ^{13}C NMR of **Boc-Cys(Bzl)-ONHS.**

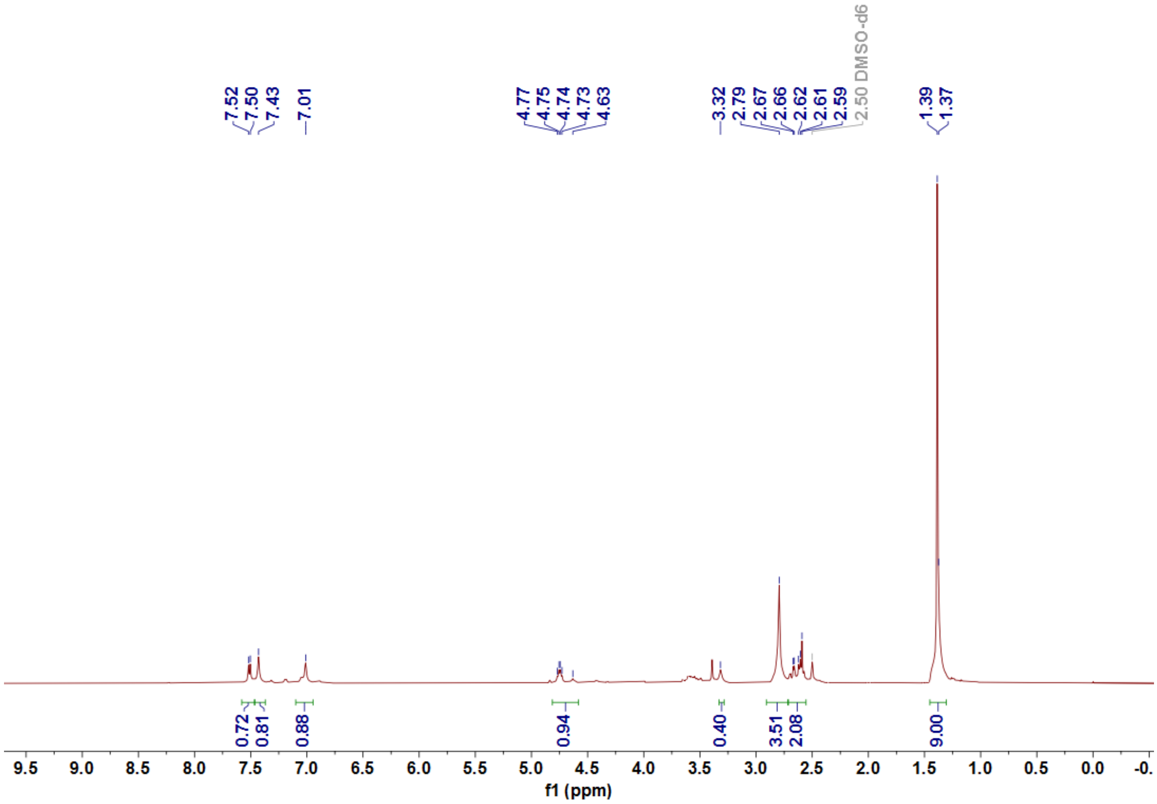

Figure S3. The ^{1}H NMR of **Boc-Asn-ONHS.**

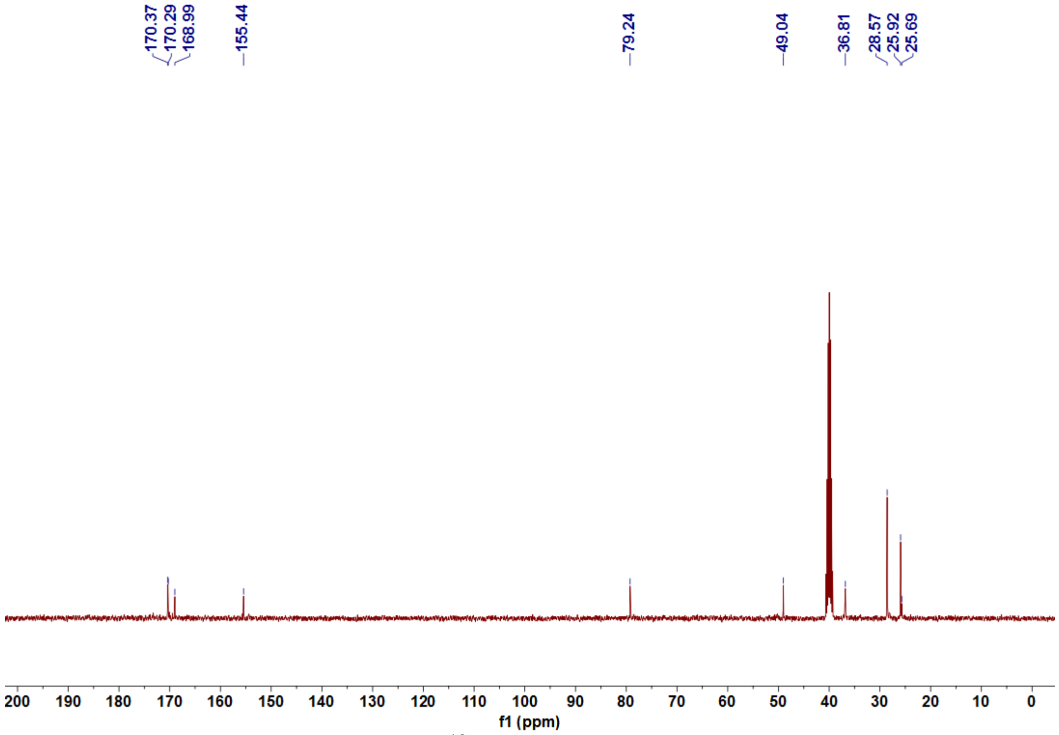

Figure S4. The ^{13}C NMR of **Boc-Asn-ONHS.**

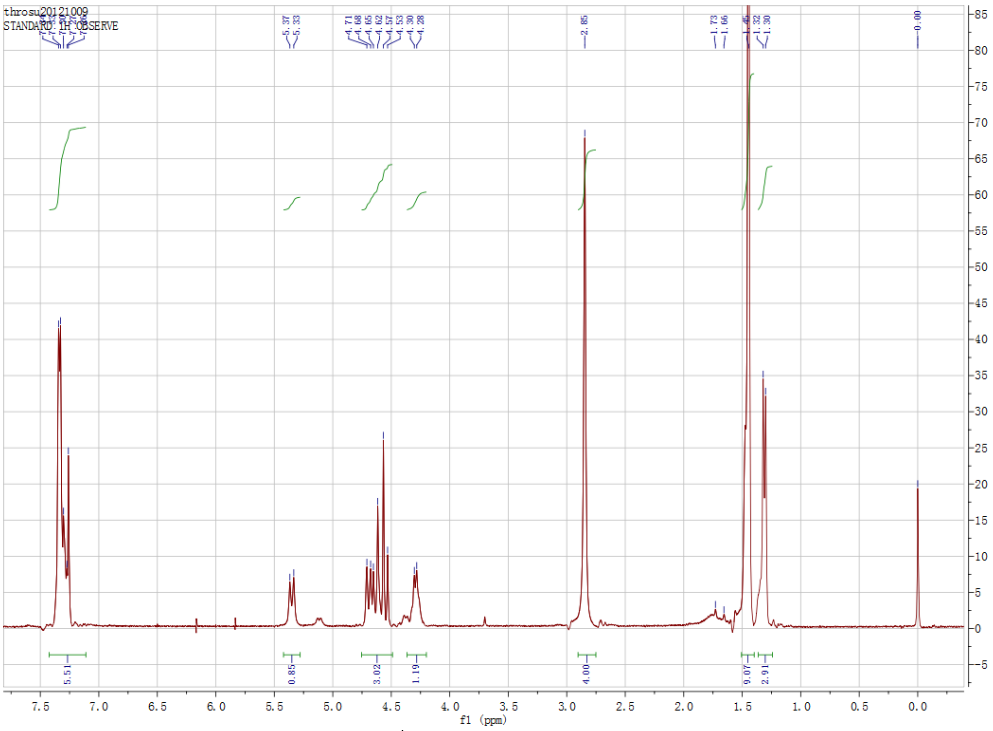

Figure S5. The ^{1}H NMR of **Boc-Thr(Bzl)-ONHS.**

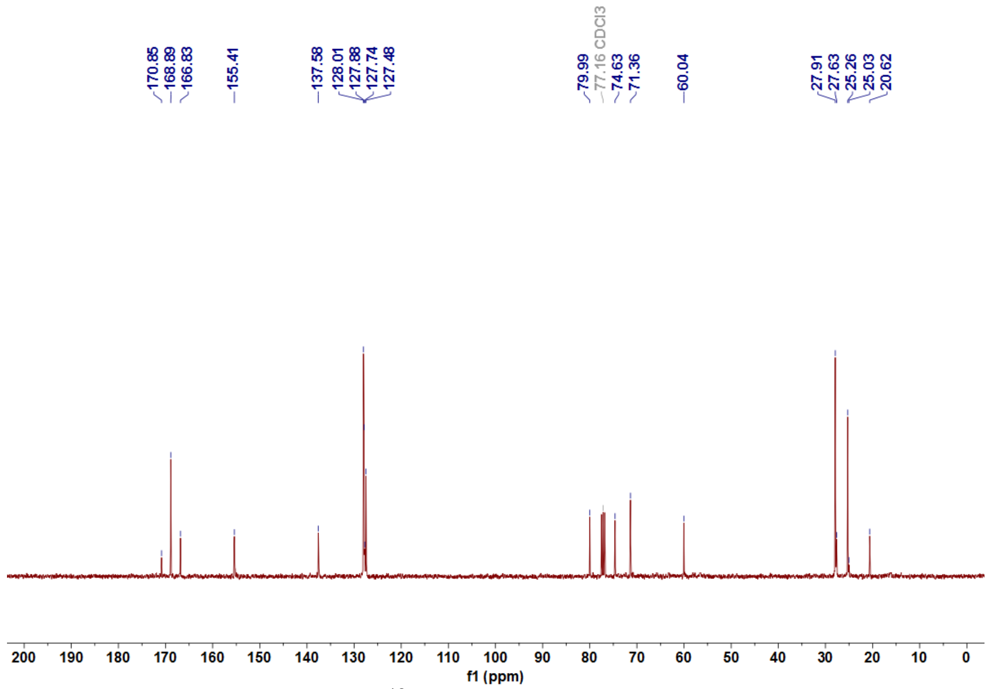

Figure S6. The ^{13}C NMR of **Boc-Thr(Bzl)-ONHS.**

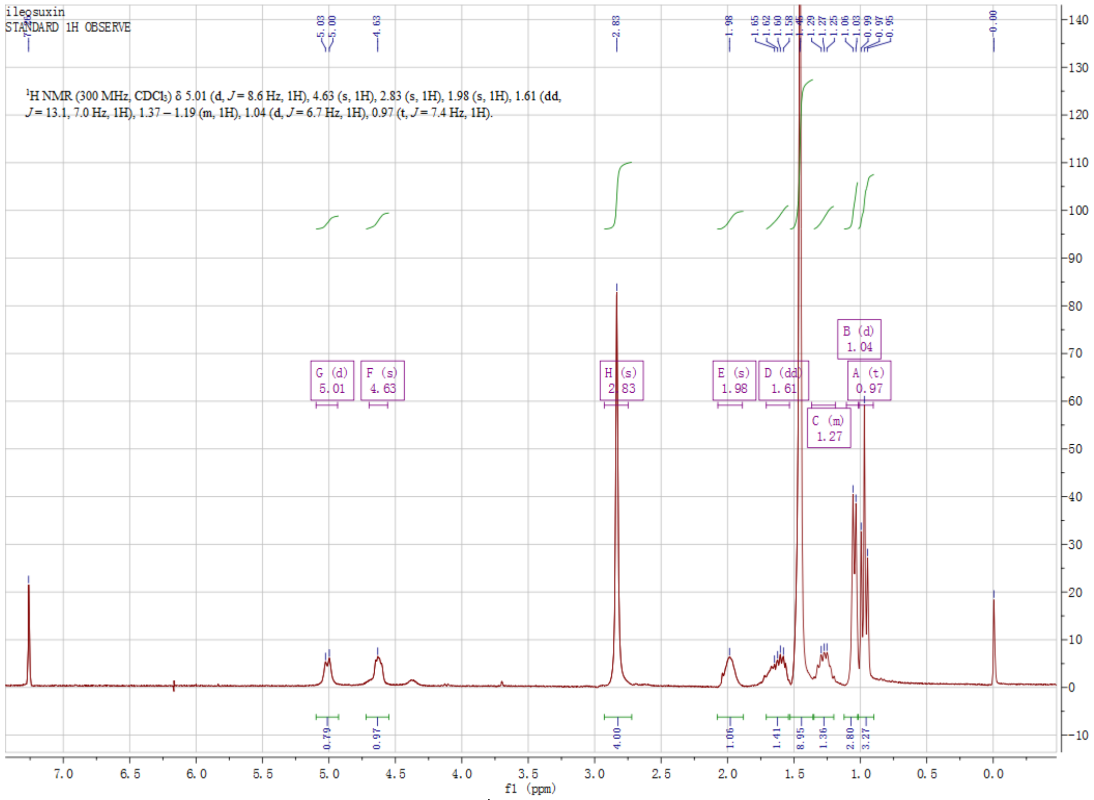

Figure S7. The ^{1}H NMR of **Boc-Ile-ONHS.**

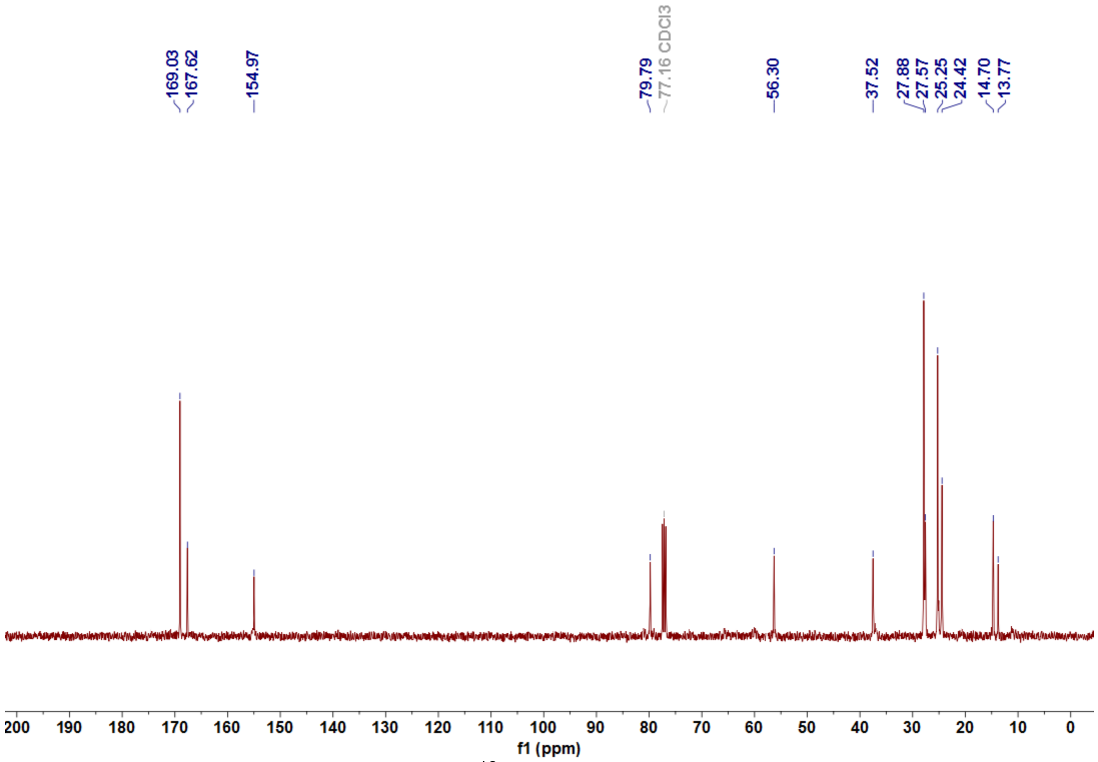

Figure S8. The ^{13}C NMR of **Boc-Ile-ONHS.**

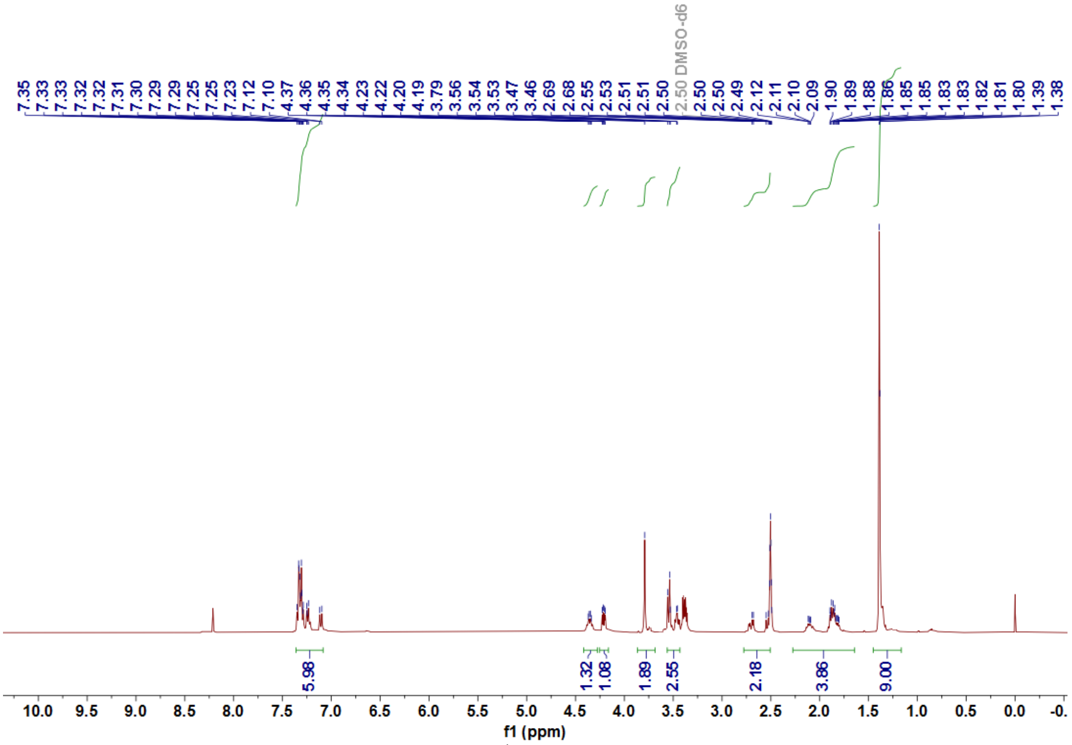

Figure S9. The ^{1}H NMR of **Boc-TM2.**

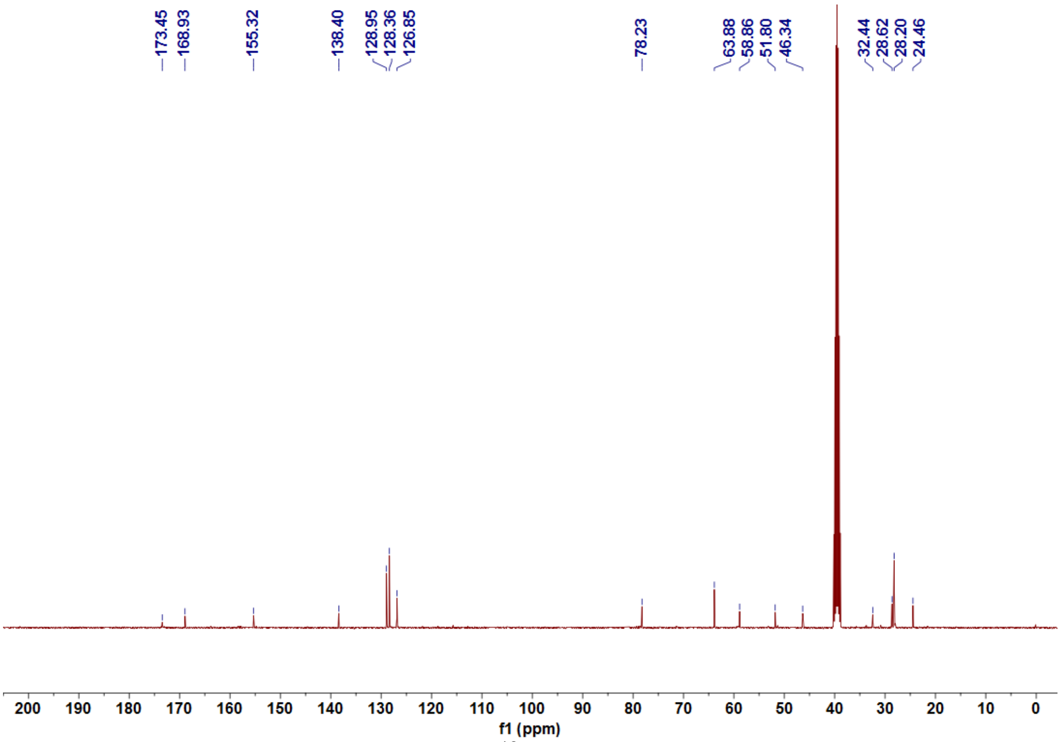

Figure S10. The ^{13}C NMR of **Boc-TM2.**

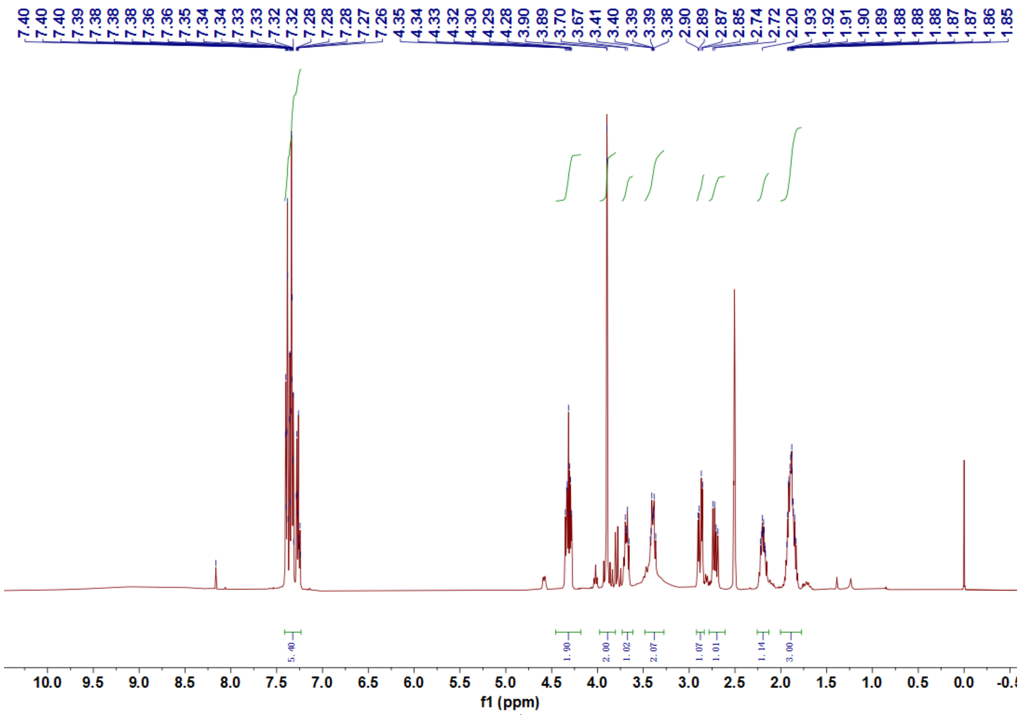

Figure S11. The ^{1}H NMR of **TM2.**

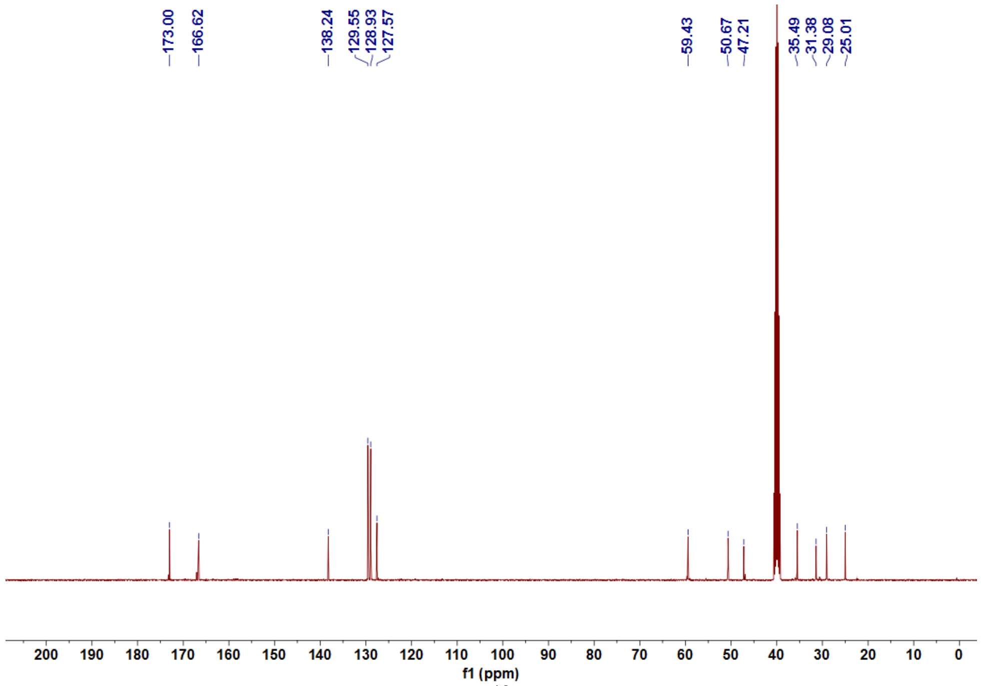

Figure S12. The ^{13}C NMR of **TM2.**

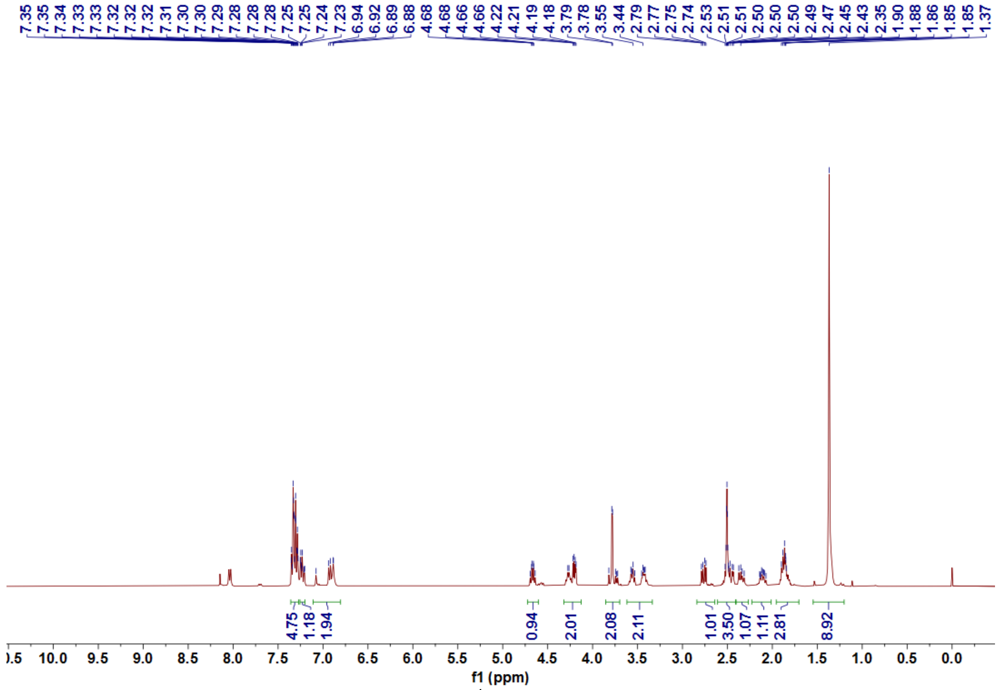

Figure S13. The ^{1}H NMR of **Boc-TM3.**

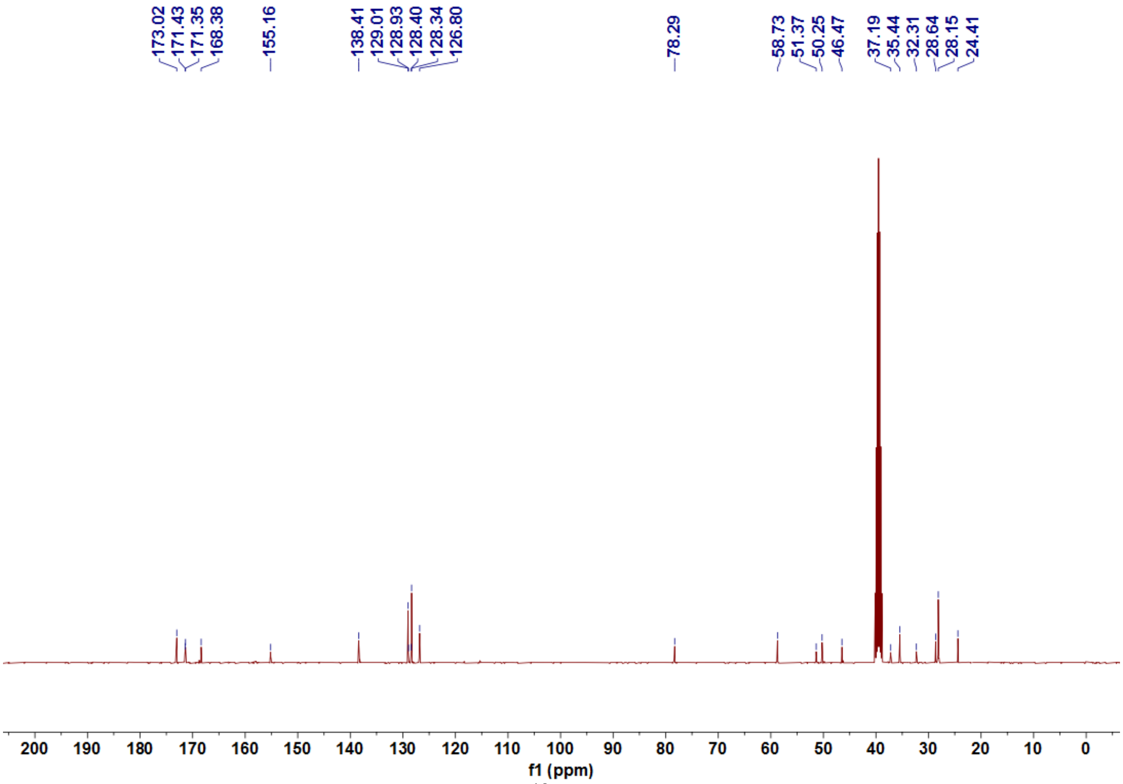

Figure S14. The ^{13}C NMR of **Boc-TM3.**

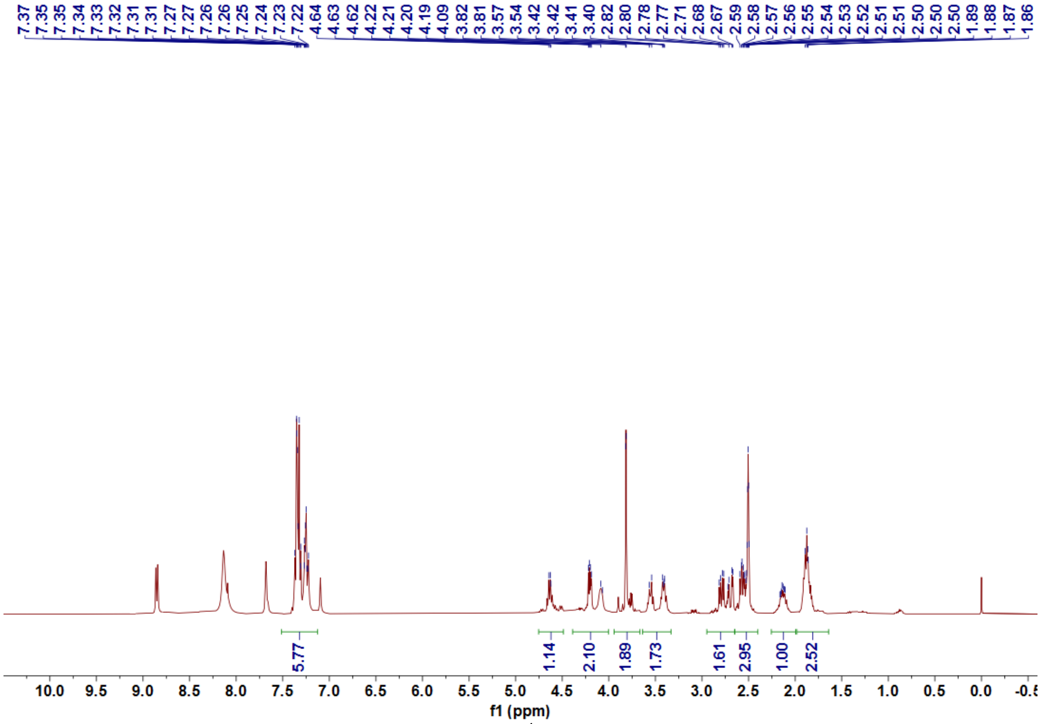

Figure S15. The ^{1}H NMR of **TM3.**

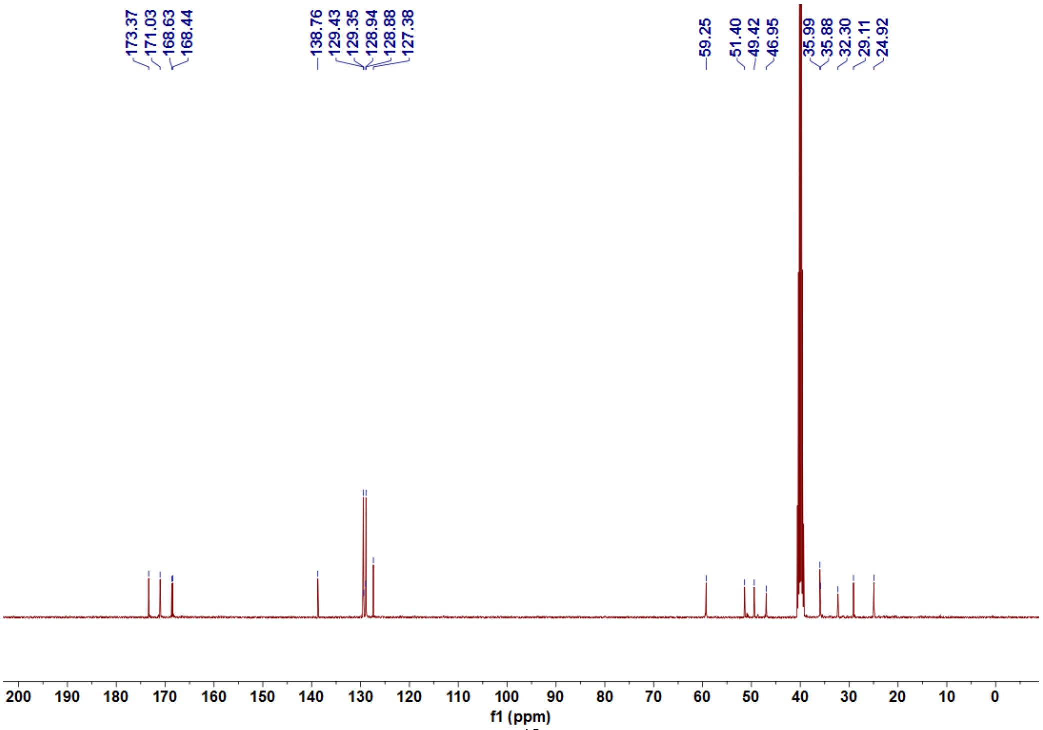

Figure S16. The ^{13}C NMR of **TM3.**

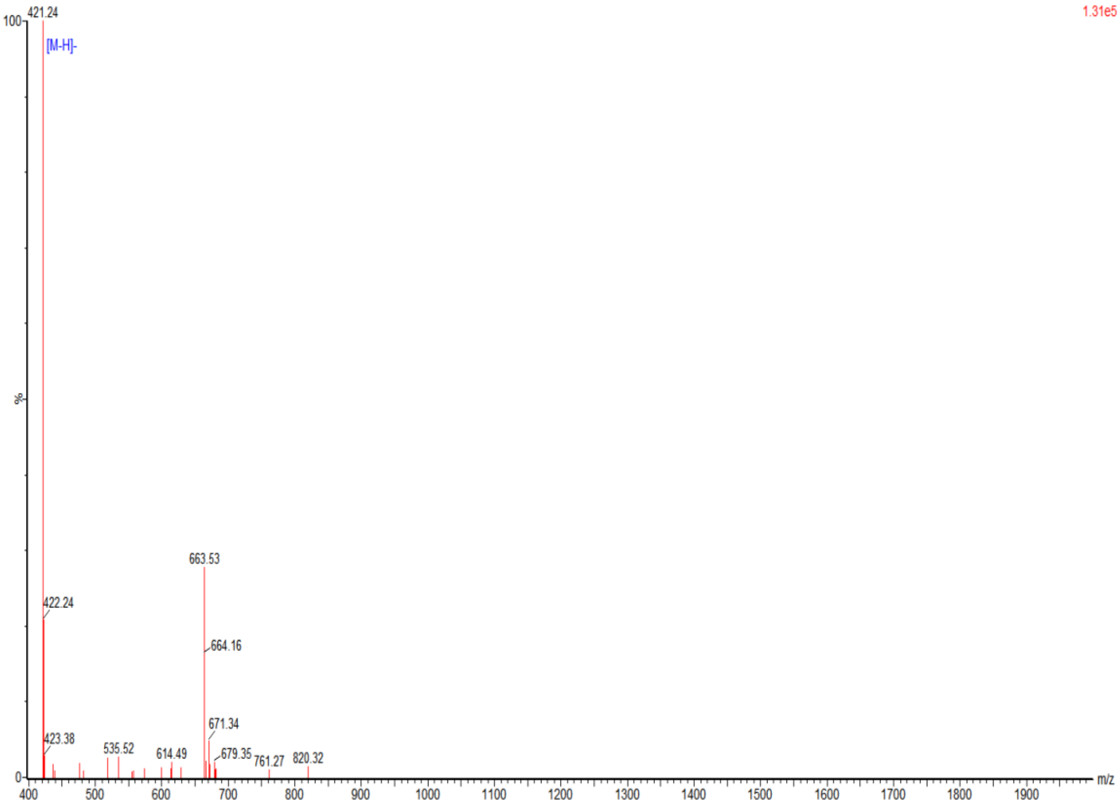

Figure S17. The MS spectrum of **TM3.**

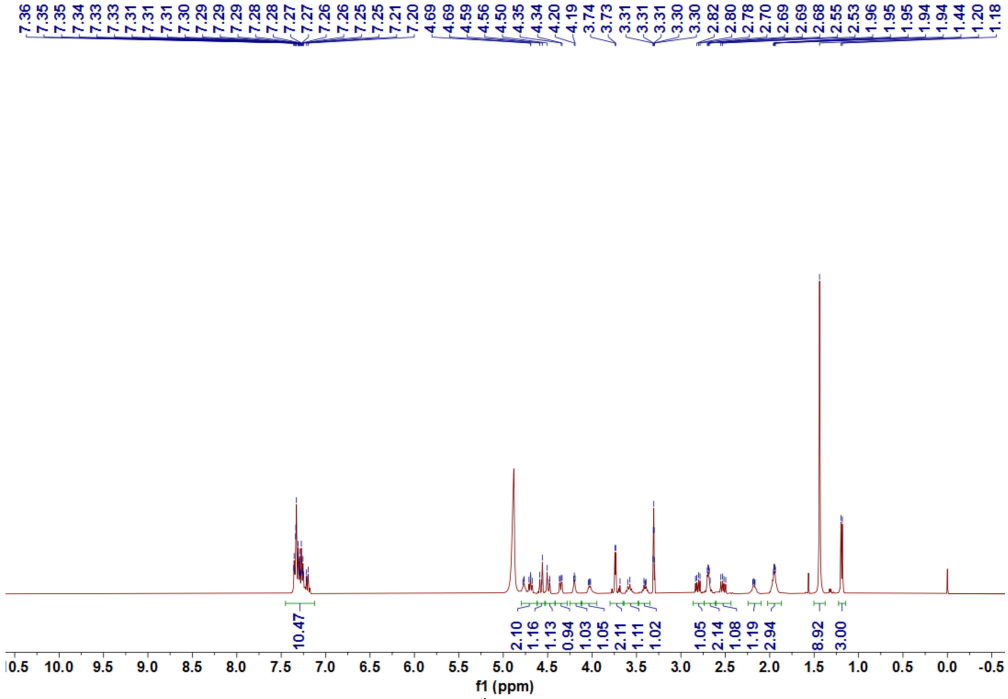

Figure S18. The ^{1}H NMR of **Boc-TM4.**

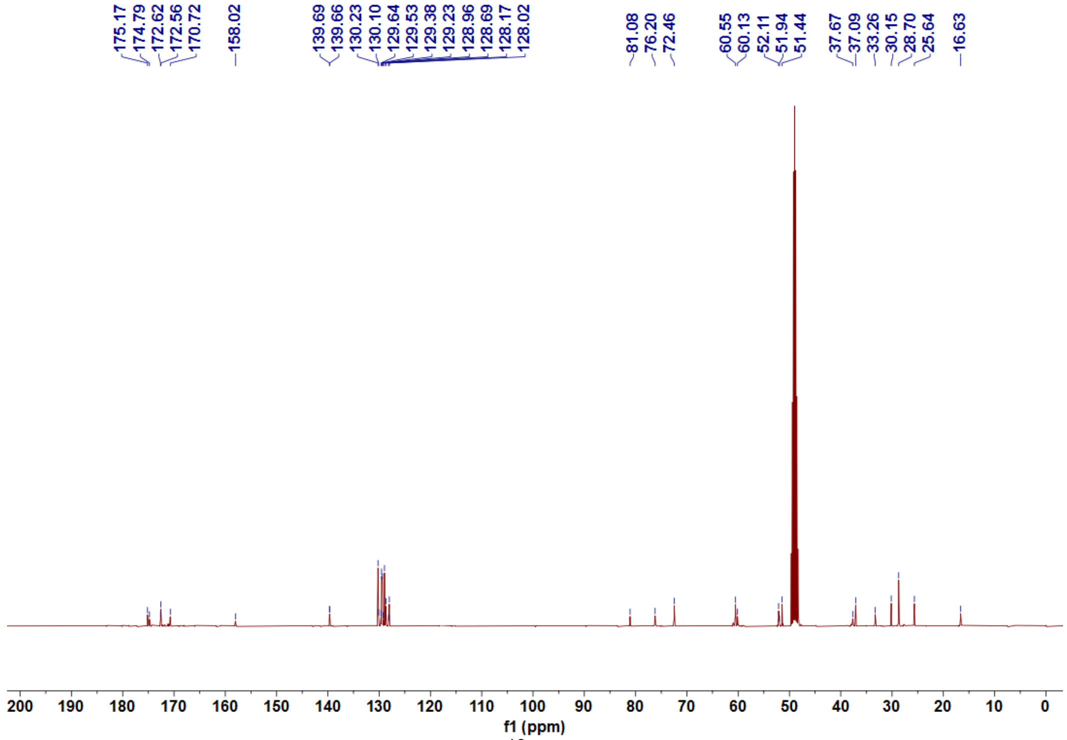

Figure S19. The ^{13}C NMR of Boc-**TM4.**

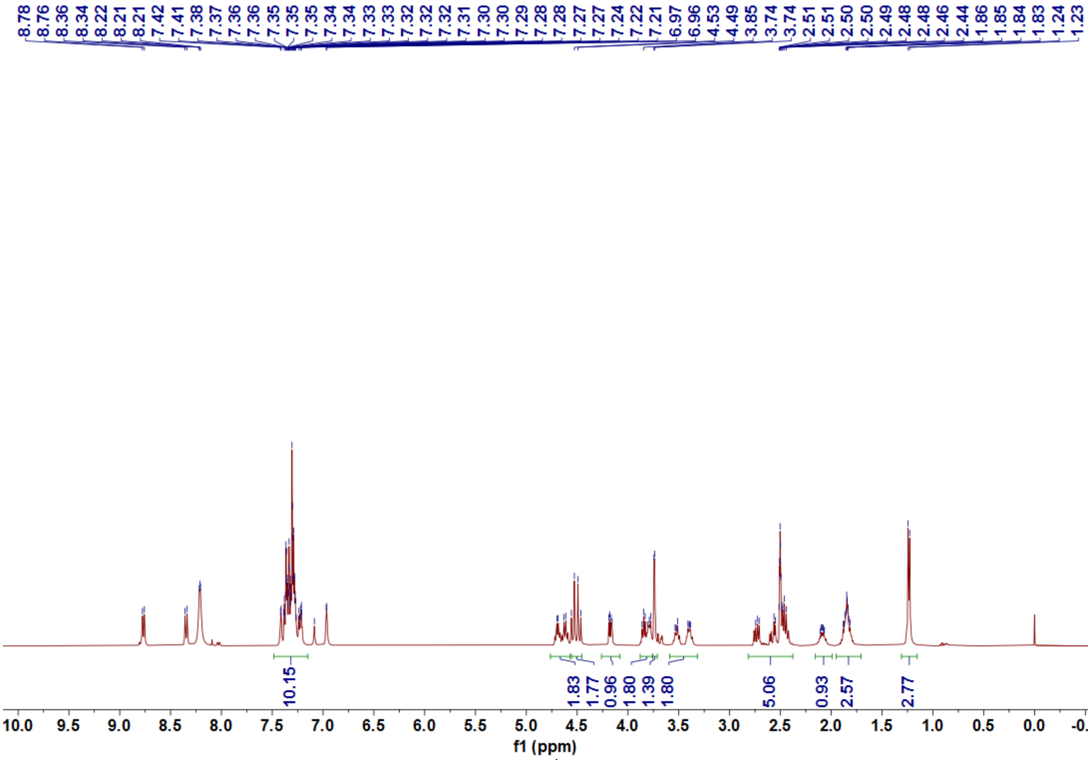

Figure S20. The ^{1}H NMR of **TM4.**

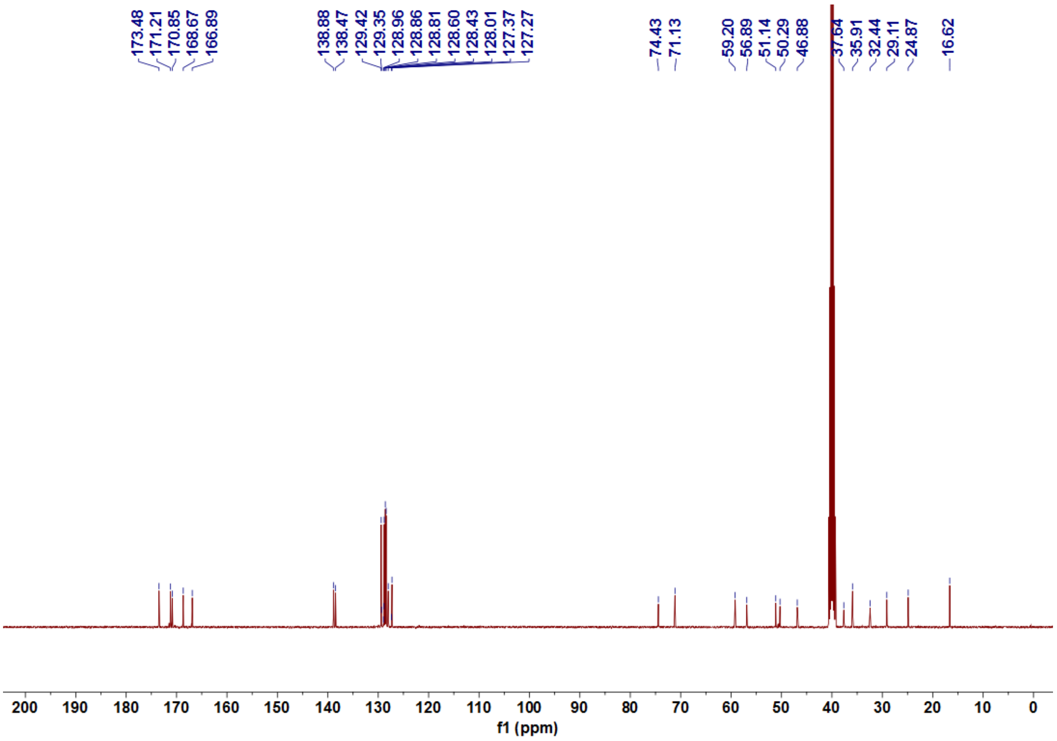

Figure S21. The ^{13}C NMR of **TM4.**

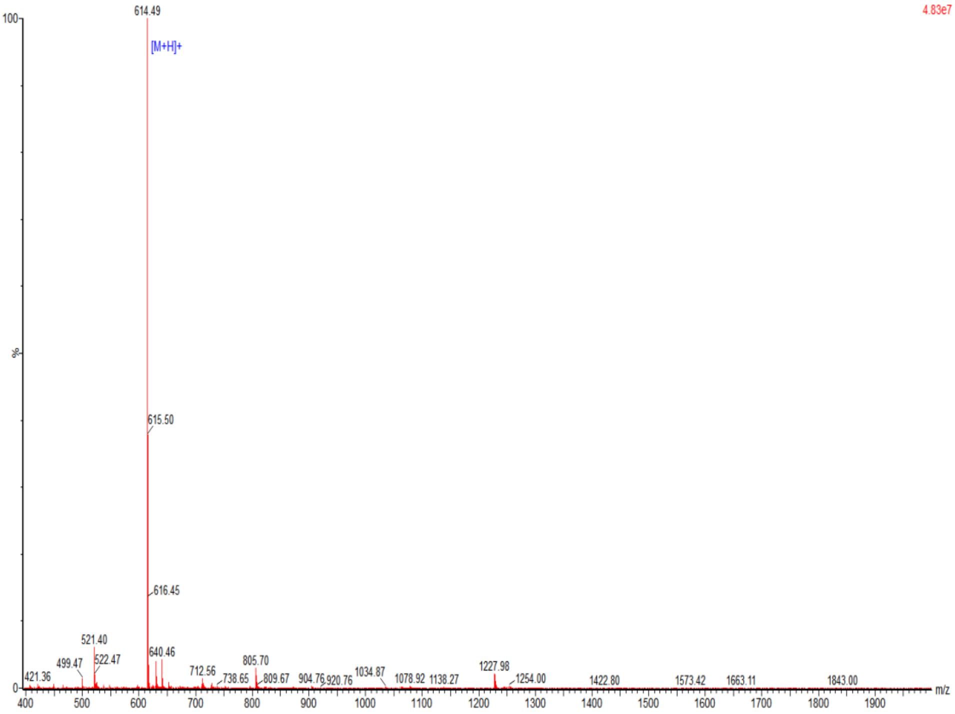

Figure S22. The MS spectrum of **TM4.**

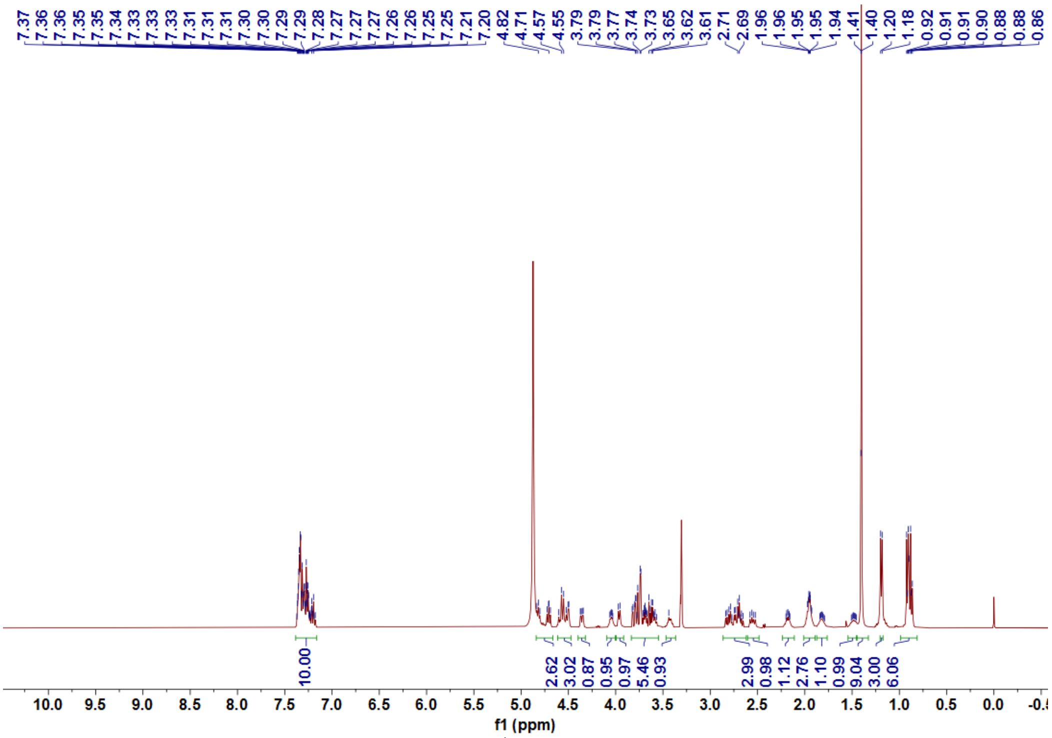

Figure S23. The ^{1}H NMR of **Boc-TM5.**

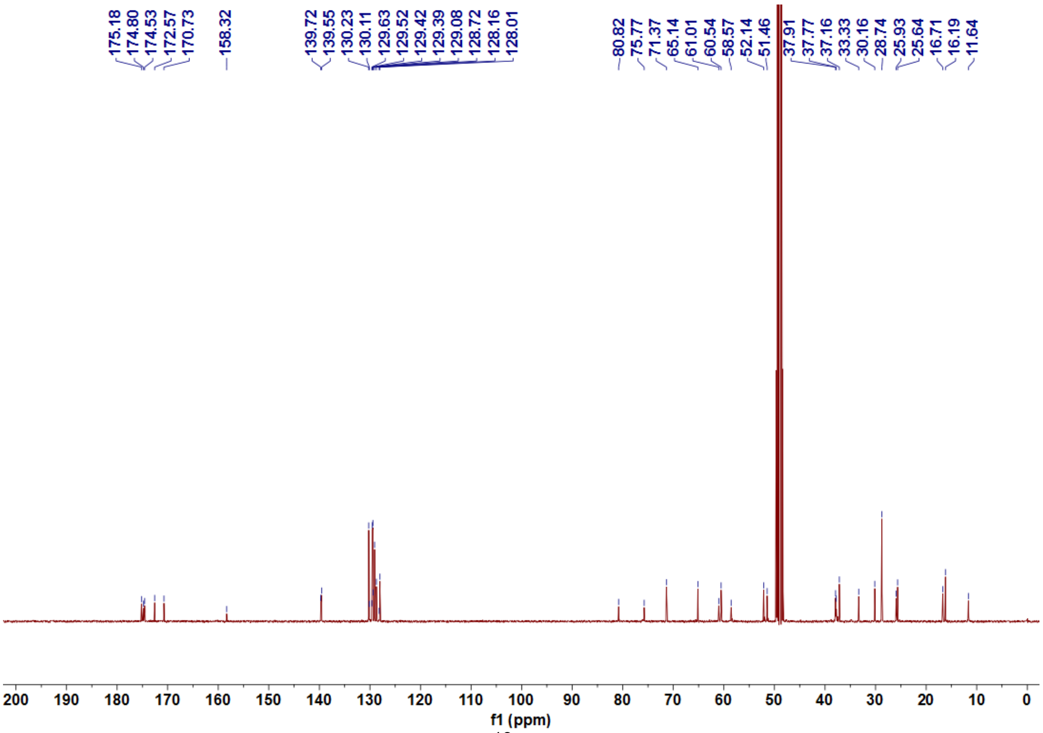

Figure S24. The ^{13}C NMR of Boc-**TM5.**

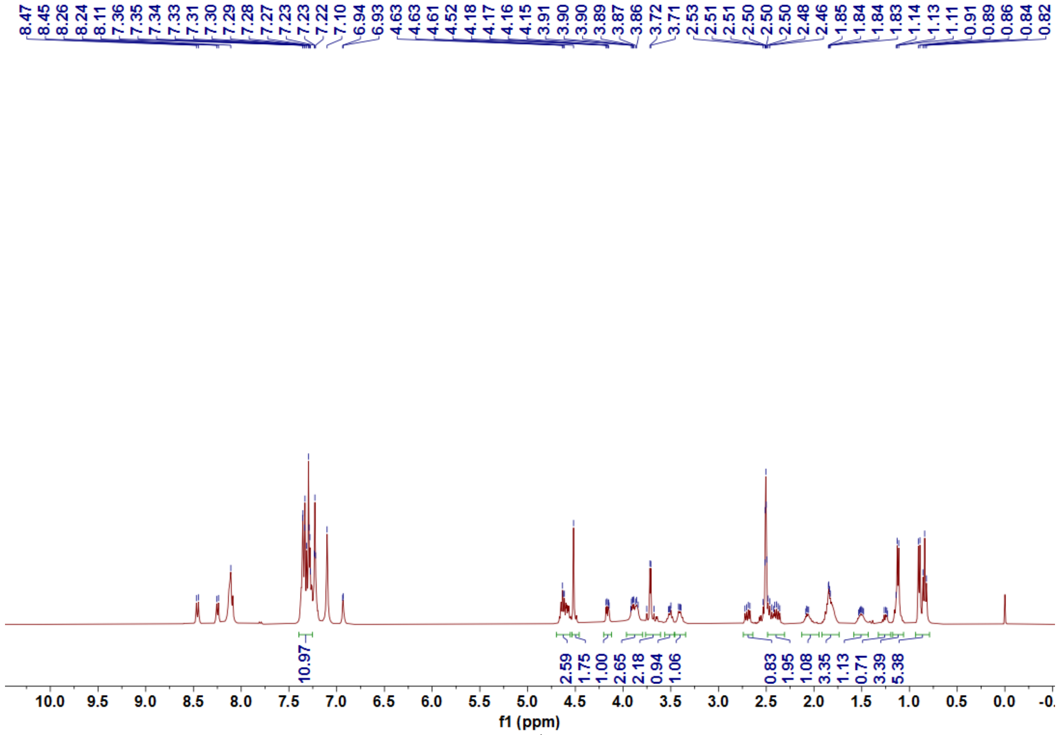

Figure S25. The ^{1}H NMR of **TM5.**

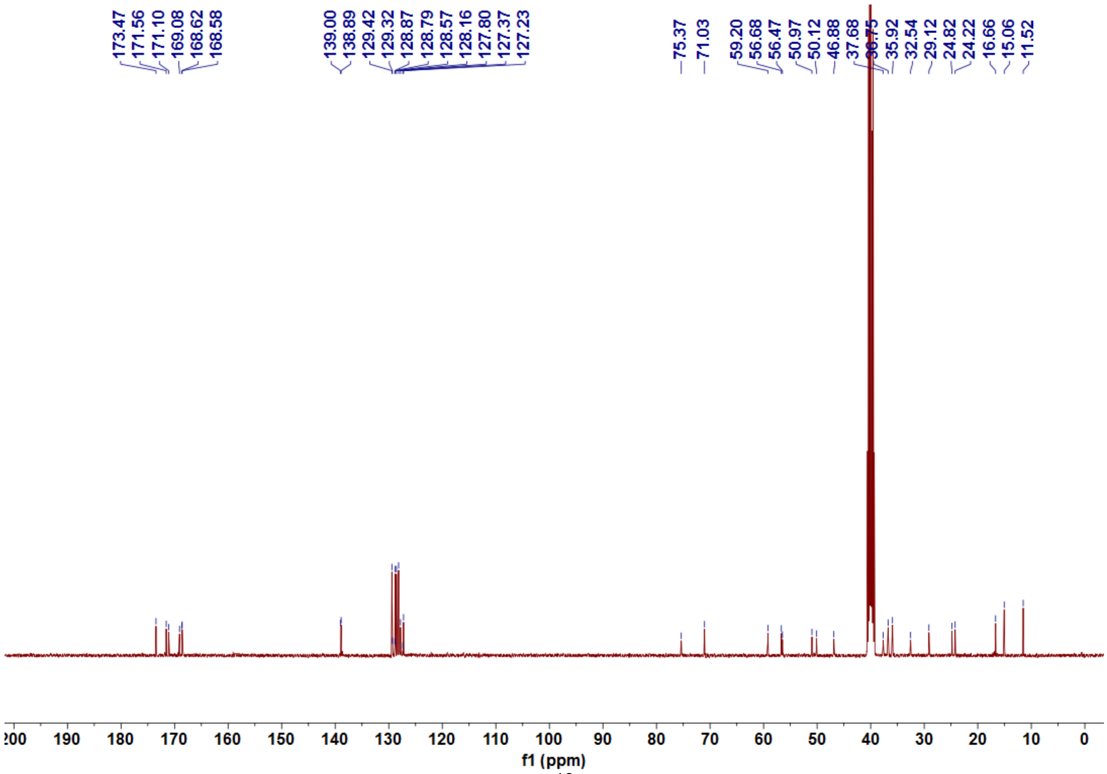

Figure S26. The ^{13}C NMR of **TM5.**

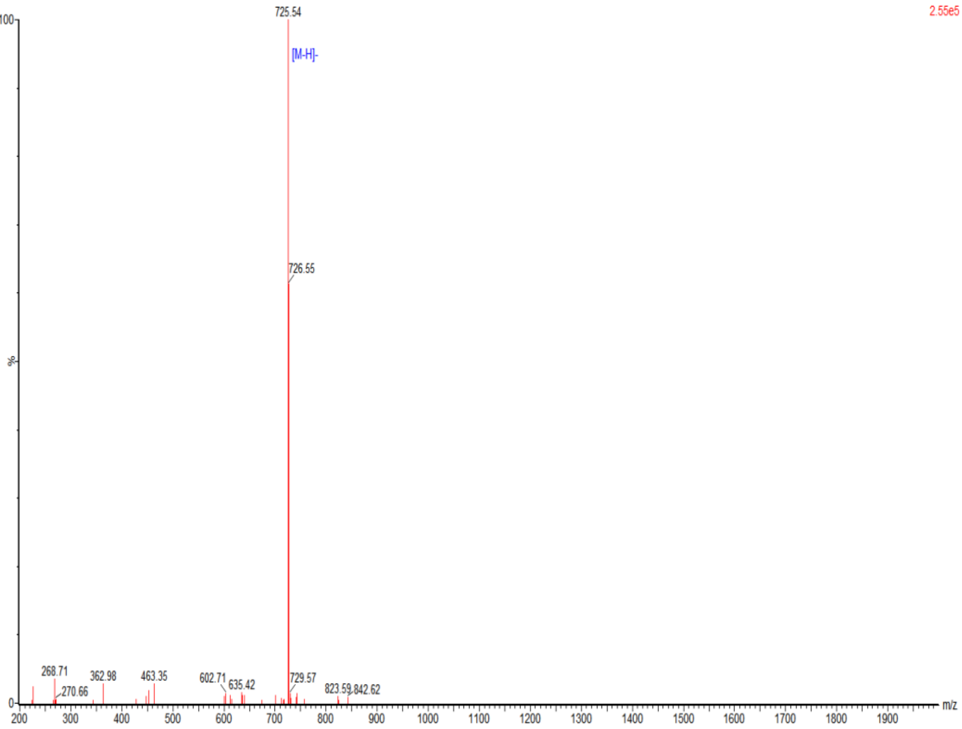

Figure S27. The MS spectrum of **TM5.**
